# Regional trade agreement burdens global carbon emissions mitigation

**DOI:** 10.1038/s41467-022-28004-5

**Published:** 2022-01-20

**Authors:** Kailan Tian, Yu Zhang, Yuze Li, Xi Ming, Shangrong Jiang, Hongbo Duan, Cuihong Yang, Shouyang Wang

**Affiliations:** 1 NCMIS, MADIS, Academy of Mathematics and Systems Science, Chinese Academy of Sciences, 100190 Beijing, China; 2 Questrom School of Business, Boston University, Boston, MA 02215 USA; 3 School of Economics and Management, University of Chinese Academy of Sciences, 100086 Beijing, China

**Keywords:** Environmental impact, Environmental economics

## Abstract

Regional trade agreements (RTAs) have been widely adopted to facilitate international trade and cross-border investment and promote economic development. However, ex ante measurements of the environmental effects of RTAs to date have not been well conducted. Here, we estimate the CO_2_ emissions burdens of the Regional Comprehensive Economic Partnership (RCEP) after evaluating its economic effects. We find that trade among RCEP member countries will increase significantly and economic output will expand with the reduction of regional tariffs. However, the results show that complete tariff elimination among RCEP members would increase the yearly global CO_2_ emissions from fuel combustion by about 3.1%, doubling the annual average growth rate of global CO_2_ emissions in the last decade. The emissions in some developing members will surge. In the longer run, the burdens can be lessened to some extent by the technological spillover effects of deeper trade liberalization. We stress that technological advancement and more effective climate policies are urgently required to avoid undermining international efforts to reduce global emissions.

## Abstract (web summary)

The Regional Comprehensive Economic Partnership (RCEP) will come into force in January 2022. Here the authors quantify ex ante economic and environmental effects following RCEP tariff reductions.

## Introduction

Regional trade agreements (RTAs) have been sweeping the world and have become ubiquitous in facilitating international trade and investment^1–3^. After an 8-year-long negotiation, the ten countries of ASEAN (Association of Southeast Asian Nations), China, Japan, South Korea, Australia, and New Zealand finally concluded the Regional Comprehensive Economic Partnership (RCEP) in November 2020 and it became the largest RTA in the world in terms of both economic size and population. According to the Schedule of Tariff Commitments in the RCEP Agreement, most trade in goods will be duty-free immediately or within ten years after the agreement enters into force. Tariff elimination in the region will reduce trade and production costs, resulting in considerable trade-creation and production-boosting effects.

However, increased international production fragmentation has raised concerns about the trade-climate dilemma (or pollution haven effect) of international trade^4–10^. That is, international trade increases global or regional emissions if developed economies with cleaner production technology and more stringent environmental policies transfer their polluting industries or production activities to developing countries, leading to emission leakages. Most RCEP member countries are typical developing economies that are less emission efficient in their manufacturing industries. In 2018, the amount of CO_2_ emitted by RCEP member countries accounted for a high share (39.1%^11^) of global CO_2_ emissions from fuel combustion. Therefore, the rapid growth of production activities and trade in an increasing number of less developed nations could impose non-negligible burdens on global and national emission mitigation. Since all the RCEP member countries have also committed under the Paris Agreement and the 2030 Sustainable Development Goals (SDG) of the United Nations^12^, quantifying both the potential economic gains and environmental burdens following RCEP is vital for balancing economic and environmental development when implementing RTAs.

There are two main strands of literature relevant to our study. The first strand of literature deals with the economic effects of RTAs by focusing on quantifying the economic welfare effects of RTAs^13–18^ and has largely neglected environmental problems. The second strand deals with the environmental side effects of international trade, which has substantially accounted ex post for the large carbon flows between countries via international trade^19–28^. The effect of international trade on emission level and intensity has also been intensively studied in the past decade^29–31^. Some studies consider the effect of international trade and foreign direct investment (FDI) when studying the energy-economy-environment relationship^32–34^. In fact, few studies have ex ante quantified the environmental effects of a trade agreement.

In this work, we estimate the carbon emission effects after evaluating the economic effects of RCEP tariff reductions. We estimate how and to what extent RCEP tariff reductions affect trade and economic welfare using a multi-sector and multi-country general equilibrium model^13^ from the perspective of global production networks or global value chains (GVCs)^35,36^. The results show that the RCEP tariff reduction will unleash trade-creation effects and improve all member countries’ economic welfares. However, we also find that complete tariff elimination within the RCEP bloc would increase the yearly global CO_2_ emissions by 3.1% if the emission intensities (CO_2_ emissions per unit of output) keep unchanged. Given that the annual average growth rate of global CO_2_ emissions from fuel combustion in the last decade was ~1.5%^11^, 3.1% represents a substantial burden on global CO_2_ emissions mitigation. The environmental burdens on some developing countries such as Vietnam and Thailand will surge. In the longer run, the learning-by-doing effects of deeper trade integration reduce the emission intensity and thereby lessen the emission burdens to some extent. However, they are not large enough to completely offset the burdens. As a result, we emphasize that technological advancements in reducing pollutant intensity are urgently required in more developing countries to offset the extra emissions caused by the RCEP. We also suggest that more effective climate policies for international trade should be designed and implemented.

## Results

### Economic effects from RCEP tariff elimination

In this subsection, we evaluate how and to what extent trade and welfare are affected by tariff reductions committed in the RCEP Agreement. Our estimation draws on three types of datasets: input–output (I.O.) tables, bilateral trade flows, and bilateral tariff data. Supplementary note 1 provides detailed descriptions of all the data used in our evaluation. We set the effectively applied ad valorem tariff rates in 2019 as the base world tariff structure before the RCEP enters into force. The effects of the RCEP tariff changes are evaluated by changing the tariff structure among RCEP member countries and leaving the tariff structure unchanged for countries outside the agreement.

**Fig. 1:**
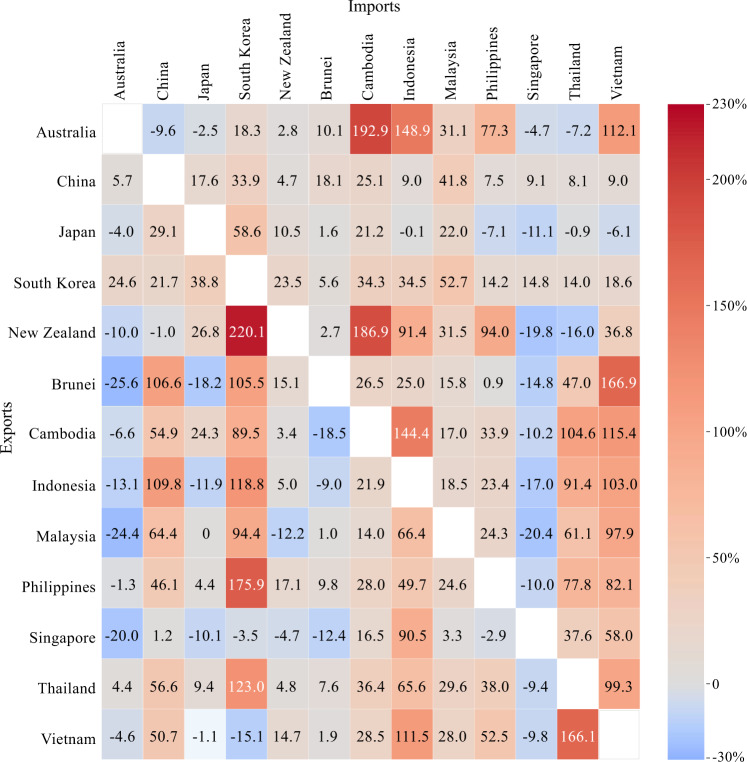
Trade effects of the RCEP tariff elimination on member countries (%). The figure presents the change rates in multilateral trade for the situation in which trade in goods among the Regional Comprehensive Economic Partnership (RCEP) members has zero tariffs. Two ASEAN (Association of Southeast Asian Nations) countries (Laos and Myanmar) are classified into the rest of the world because input–output tables for Laos and Myanmar are not available. We thus cannot provide results for these two countries. According to the Schedule of Tariff Commitments in the RCEP agreement (Supplementary note 1), most tariff reductions will be implemented within ten years, which implies that most of the impacts will be achieved within ten years. The results for the cases in which the tariffs decline to the committed level in years 1, 5, 10, and 20 after the RCEP enters into force are provided in Supplementary Tables 4–7. The results for any other years are also available upon request.

Figure 1 presents the aggregate changes in multilateral trade for RCEP member countries when tariffs within the bloc decline to zero after the agreement enters into force. Unsurprisingly, tariff elimination will gradually unleash the agreement’s trade-creation effect and substantially strengthen the trade linkages between these countries before the RCEP. We also observe that the trade effects vary across members. First, the RCEP will significantly increase trade between China, Japan, and South Korea. Specifically, China’s exports to Japan and South Korea will increase by 17.6% and 33.9%, and Japan’s exports to China and South Korea will increase by 29.1% and 58.6%, respectively. Second, the RCEP will boost more trade for some ASEAN economies. For example, Indonesia’s exports to China, South Korea, Thailand, and Vietnam will increase quite markedly by 109.8%, 118.8%, 91.4%, and 103.0%, respectively. Similar effects will occur for certain other ASEAN economies, such as Malaysia, the Philippines, Thailand, and Vietnam. It is apparent that tariff elimination in the RCEP bloc will improve South-North and South-South trade. The equilibrium model indicates that the trade effects are determined by the magnitude of tariff reduction, export bundles, trade elasticity, and the intermediate input structure for the production of each sector in each country. The divergent effects shown in Fig. 1 are the complete results of these differently weighted factors. Another observation is that some bilateral trade (e.g., the exports of Singapore to Australia and Japan) will decline after the RCEP enters into force. This negative effect arises from trade diversion. For example, Singapore was already an almost entirely free-trade country before the RCEP. The RCEP tariff reduction substantially reduces the costs of trade with other members and raises their relative competitiveness. This process facilitates trade diversion from Singapore.

Table 1 presents the results of welfare effects for RCEP members. It shows that all members benefit from the RCEP tariff reductions, with most small countries gaining more than large ones. The first column presents that all RCEP members’ real wages will increase, with Cambodia increasing the most. The second column shows that the welfare (the summation of labor income, tariff revenues, and trade deficits) of Vietnam, Cambodia, and Singapore will increase the most, by 15.6%, 8.5%, and 3.8%, respectively. The effects for large economies such as China and Japan are smaller. This result supports the convergence theory that trade liberalization enables small developing economies to develop faster than more developed countries.

**Table 1:** Welfare effects of the RCEP tariff elimination on member countries (%).

Members	Real wage	Welfare				
		Total	Volume of trade		Terms of trade	
			RCEP	RoW	RCEP	RoW
Australia	0.3	0.2	0.0	0.0	0.1	0.1
China	0.4	0.4	0.2	0.0	0.1	0.1
Japan	0.3	0.3	0.1	0.0	0.1	0.1
South Korea	2.4	3.3	4.5	−0.5	−0.4	−0.3
New Zealand	0.4	0.5	0.0	0.0	0.3	0.2
ASEAN member						
Brunei	0.5	0.6	0.0	0.0	0.5	0.1
Cambodia	11.9	8.5	5.1	−0.5	1.8	2.1
Indonesia	0.9	0.8	0.4	0.0	0.2	0.2
Malaysia	4.7	1.3	1.2	−0.1	0.1	0.1
Philippines	2.0	0.5	0.4	0.0	0.0	0.1
Singapore	3.5	3.8	0.0	0.0	1.7	2.1
Thailand	3.2	1.8	2.5	−0.2	−0.4	−0.1
Vietnam	5.6	15.6	10.2	−0.4	3.1	2.7

Note: The table presents the changes in real wage and welfare for the Regional Comprehensive Economic Partnership (RCEP) members if trade in goods among the members becomes duty-free. We do not have specific results for two ASEAN (Association of Southeast Asian Nations) countries (Laos and Myanmar) due to data unavailability. The total welfare effects are decomposed into the volume of trade effect and terms of trade effect, which are further decomposed separately into the results of trade with RCEP members (columns 3 and 5) versus trade with the rest of the world (RoW, columns 4 and 6).

To reveal how each member’s welfare is improved, we decompose the total welfare effects into the volume of trade effect (columns 3 and 4) and terms of trade effect (columns 5 and 6), which are further decomposed separately into the results of trade with RCEP members versus trade with other economies outside the RCEP Agreement (the rest of the world, RoW). Columns 3 and 4 show that trade with RCEP members rather than the RoW is the most important contributor to the increase in all members’ volume of trade. Comparing with the results in columns 5 and 6, we can find that creating more trade within the RCEP bloc also makes the most significant contribution to the increase in welfare for member countries—South Korea, Cambodia, Malaysia, the Philippines, Thailand, and Vietnam. In addition, trade reductions with the RoW generate slightly negative welfare effects for most members. The reason for such negative effects stems from the RCEP diverting trade from non-RCEP member countries.

Columns 5 and 6 show that the aggregate terms of trade for almost all members improve, whereas South Korea’s and Thailand’s terms of trade deteriorate slightly. This differential performance can be attributed to the divergent changes in the export prices of each country, as terms of trade compare the price of a country’s export with the price of its import. The model shows that export prices are determined by the unit costs of input bundles, namely, the combination of labor costs (i.e., wages) and the prices of intermediate inputs. Column 1 shows that the RCEP will increase the real wages of all members, which increases export prices. However, other things being equal, the prices of intermediate inputs will decline with tariffs on imported intermediates. Such effects can further be propagated through input–output linkages. As a result, the change in the prices of intermediate inputs decreases export prices. Ultimately, for most RCEP members, the increase in real wages is larger than the decrease in the prices of intermediate inputs, which results in a positive effect on terms of trade. For other economies, the contrary is the case.

Supplementary Tables 8 and 9 provide the welfare effects of RCEP tariff reductions on economies outside this agreement. The effects are twofold. On the one hand, as explained above, the agreement generates trade diversion towards RCEP members. On the other hand, in an era of increased international fragmentation, the production activities of trade products in countries outside the RCEP require intermediate inputs from RCEP members. The decreased prices of such intermediate products due to RCEP tariff reductions also change the production costs, export prices, and terms of trade in economies outside the RCEP. As a result, the effects are negative for some non-RCEP economies but positive for others. However, the impact is minimal.

**Fig. 2:**
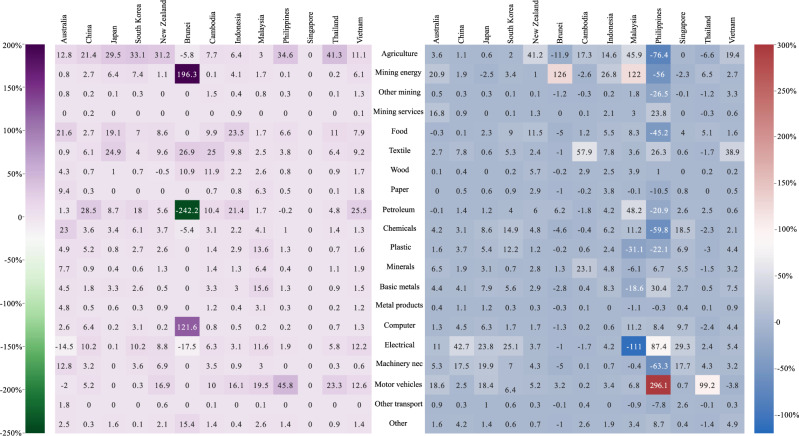
Sectoral contribution to the welfare changes in RCEP members (%). The left graph presents the sectoral contribution to the aggregate changes in the volume of trade in the case that all trade in goods among the Regional Comprehensive Economic Partnership (RCEP) members are duty-free, while the right graph presents the results for terms of trade. For a country, the column-wise summation of all sectors’ contributions equals 100%. The description of the tradable sectors is provided as follows (Supplementary Table 1). Agriculture: agriculture, forestry, and fishing; Mining energy: mining and extraction of energy-producing products; Other mining: mining and quarrying of non-energy-producing products; Mining service: mining support service activities; Food: food products, beverages, and tobacco; Textile: textiles, wearing apparel, leather, and related products; Wood: wood and products of wood and cork; Paper: paper products and printing; Petroleum: coke and refined petroleum products; Chemicals: chemicals and pharmaceutical products; Plastic: rubber and plastic products; Minerals: other nonmetallic mineral products; Basic metals; Metal products: Fabricated metal products; Computer: computer, electronic, and optical products; Electrical: electrical equipment; Machinery nec: machinery and equipment, nec; Motor vehicles: motor vehicles, trailers, and semi-trailers; Other transport: other transport equipment; Other: other manufacturing, repair, and installation of machinery and equipment.

For each RCEP member, we calculate the sectoral contribution to its aggregated change in volume of trade and terms of trade. First, as shown in Fig. 2, the contribution varies considerably across countries and sectors. For example, mining is the sector with the most significant contribution to the change in Australia’s terms of trade, whereas electrical equipment is the most significant contributor for China and Japan. Second, agriculture (and food products for some countries) significantly contributes to changes in the volume of trade in many countries, including China, Japan, South Korea, New Zealand, the Philippines, and Thailand. The reason is that agriculture is strongly protected in most countries, as the current tariffs are relatively high. For example, the average tariffs applied by Japan to its imported food products and agriculture in 2019 were 10.74% and 5.84%, respectively, which were the largest and second-largest import tariffs among all Japanese goods sectors. Agriculture (and food products) is a homogeneous goods sector with high-import tariff trade elasticity. A slight reduction in the tariffs for this sector can improve the trade volume considerably because it is relatively easy to change suppliers. Petroleum in some countries is similarly affected for similar reasons.

Another notable observation is that a handful of sectors—electrical equipment, machinery and equipment, and motor vehicles—explain a high proportion of the changes in terms of trade in many countries (China, Japan, South Korea, and some ASEAN economies). This is the combined result of tariff reduction, the share of intermediate inputs required in the production process, and intersectoral linkages. Although the tariff reductions in these technology-intensive sectors are not the largest, they are considerable for some countries. More importantly, these sectors use a considerably larger share of intermediate inputs in production than other sectors. They also have stronger input–output linkages with other sectors. Therefore, a decrease in the unit production costs in these sectors has a larger multiplicative effect and thus a larger impact on terms of trade.

### Carbon-emission burdens of RCEP tariff reductions

The synergy of tariff elimination within the RCEP bloc substantially reduces the costs of intraregional trade and production and thus increases the outputs of the member countries. As a result, carbon emissions will also increase significantly if the emission intensities (emissions per unit of output, tonnes CO_2_ per US dollar in 2015) do not decrease enough to offset the extra emissions caused by the increase in production outputs. Assuming the emission intensities of all countries stay at the same level as that in the year 2015, the global CO_2_ emissions from fuel combustion would increase by 251.4 million tonnes (Mt; 0.8% compared to the amount in 2018) when the tariff structure changes to that specified for the first year after the RCEP enters into force. When the tariffs continue to decline to the level in the 5th and 10th years and then to zero, global CO_2_ emissions will increase by 463.7 Mt (1.4%), 756.4 Mt (2.3%), and 1046.5 Mt (3.1%), respectively. Recalling that global CO_2_ emissions grew at an annual average rate of 1.5%^11^ in the last decade, these results indicate substantial burdens on carbon-emission mitigation.

**Fig. 3:**
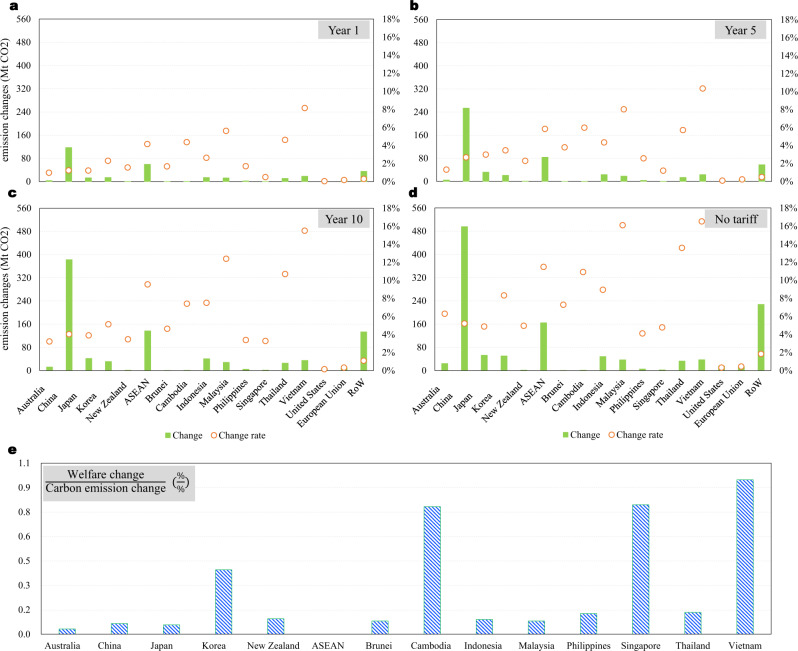
CO_2_ emission burdens of RCEP’s tariff reductions. **a**, **b**, **c** present the changes and change rates in the amount of CO_2_ emissions emitted by different economies for the cases, in which the tariffs within the Regional Comprehensive Economic Partnership (RCEP) bloc declined to the level in year 1, 5, and 10 after the RCEP enters into force, respectively. **d** presents the corresponding results for the case in which trade in goods among RCEP members is ultimately duty-free. **e** gives the ratio of economic welfare change to the CO_2_ emission change rate for RCEP members (Supplementary Table 10). ASEAN denotes the Association of Southeast Asian Nations. Here, Laos and Myanmar are still classified into the rest of the world (RoW) for the same reason as given in the note of Fig. 1.

The increased CO_2_ will be emitted mainly by RCEP member countries. In the situation of all trade in goods in the RCEP region becoming duty-free, the production of RCEP members would increase emissions by 789.1 Mt CO_2_ (Fig. 3d), accounting for 75.4% of the increased global emissions. Among, Mainland China will be the largest contributor in terms of absolute value (495.7 Mt CO_2_, 47.4% of the increased global emissions), followed by the ASEAN economies (164.7 Mt CO_2_, 15.7%) and Japan (52.7 Mt CO_2_). In terms of magnitude, Vietnam will increase the most (16.5%), followed by Malaysia (16.1%) and Thailand (13.6%). The results indicate that the emission intensities in these countries must decrease by the same magnitude to ensure that the CO_2_ emissions do not increase. The magnitude of the decrease should be larger if countries aim to reduce their emissions. Such an ambitious target could be a non-negligible burden, especially for some developing ASEAN economies.

In Fig. 3e, we also present the ratio of welfare change to the CO_2_ emission change rate for RCEP members. The ratio gives the welfare gains at the cost of a 1% increase in carbon emissions. Vietnam, Singapore, and Cambodia are the greatest gainers in this ratio, whereas China and Japan rank at the lower end.

The increased carbon emissions are driven by the rise of production for both domestic expenditures and trade. As mentioned above, the RCEP tariff reductions bring mainly trade-creation effects within the RCEP bloc and cause trade diversion between RCEP members and non-RCEP economies. An RCEP member may emit more CO_2_ in its increased trade with other RCEP members and reduce carbon emissions because of its decreased trade with non-RCEP economies. We employ the environmentally extended inter-country input–output (ICIO) model to account for the comprehensive carbon-emission changes due to trade changes. The results show that trade changes in the case of all trade in goods within the RCEP bloc becoming duty-free would increase CO_2_ emissions for China, the ASEAN countries, South Korea, Japan, Australia, and New Zealand by 130.2 Mt, 70.4 Mt, 27.2 Mt, 22.5 Mt, 4.8 Mt, and 0.5 Mt, respectively.

We also find that trade changes slightly increase the amount of CO_2_ (61.2 Mt) emitted by RoW (non-RCEP economies). The emission effects of RCEP on non-RCEP economies are twofold. On the one hand, RCEP tariff reductions reduce the direct exports of some non-RCEP economies to RCEP members for reasons we discussed in the previous section, which will reduce the emissions of some non-RCEP economies. On the other hand, the increased production for trade within the RCEP bloc requires more intermediate inputs from some economies outside the RCEP. The economic activities associated with the increased production of such intermediate products generate more CO_2_ emissions in non-RCEP economies. Due to the increased international production fragmentation, the emissions generated by these indirect linkages can be substantial. The fact that the RCEP increases overall emissions by economies outside indicates that the indirect effects are larger than the direct effects.

**Fig. 4:**
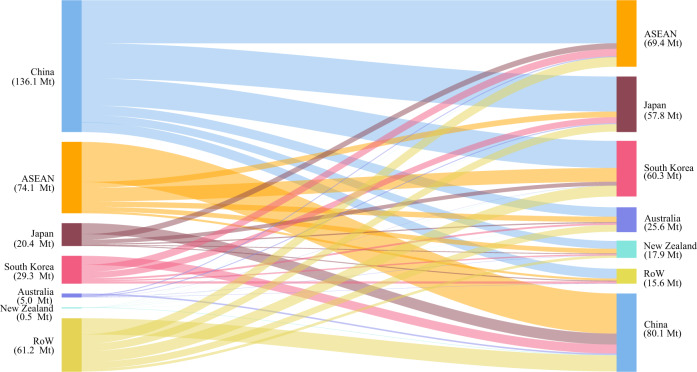
Increased bilateral CO_2_ emission flows in trade. The graph distinguishes seven regions as the origin (the left) and destination (the right). These are China, the ASEAN (Association of Southeast Asian Nations, not including Laos and Myanmar), Japan, South Korea, Australia, New Zealand, and the rest of the world (RoW). The graph presents the increased amount of CO_2_ emitted in the region of origin for its production of exports to a destination (Supplementary Table 11).

Figure 4 visualizes increased CO_2_ emissions due to changes in bilateral trade flows, which tells us who emits increased CO_2_ for whom. It shows the amount of CO_2_ emitted by a region of origin for the production of its increased exports to the destination. The largest flow is 44.3 Mt CO_2_ for China’s increased exports to the ASEAN countries. Other large flows include emissions for the increased exports of the ASEAN countries to China (40.2 Mt CO_2_), China to Japan (36.1 Mt CO_2_), and China to South Korea (27.8 Mt CO_2_). Figure 4 also shows that the increase in exports of Japan (20.4 Mt CO_2_) and South Korea (29.3 Mt CO_2_) generates a relatively small increase in the CO_2_ emissions of these countries. However, their imports generate considerable increases in CO_2_ emission (57.8 and 60.3 Mt CO_2_) in the source countries. The results indicate that developing RCEP members, including China and some ASEAN economies will increase CO_2_ for the developed members.

**Fig. 5:**
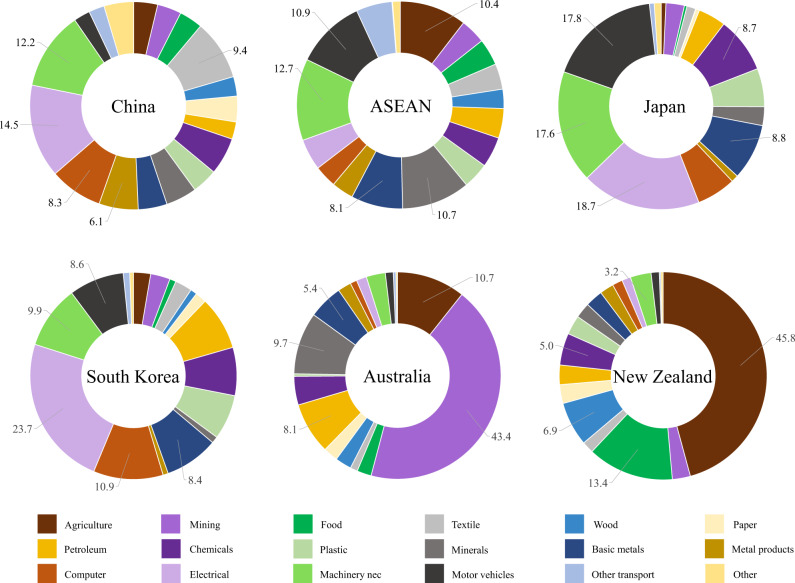
Sectoral contribution to CO_2_ emissions in trade. The pie charts present for the Regional Comprehensive Economic Partnership (RCEP) members the sectoral contribution to their CO_2_ emission changes caused by trade changes (Supplementary Table 12). ASEAN denotes the Association of Southeast Asian Nations, but Laos and Myanmar are not included due to data unavailability. The sector classification is the same as in Fig. 2, but here we aggregate the three mining-related sectors into one mining sector. Agriculture: agriculture, forestry, and fishing; Mining: mining and extraction of energy-producing products, mining and quarrying of non-energy-producing products, mining support service activities; Food: food products, beverages, and tobacco; Textile: textiles, wearing apparel, leather and related products; Wood: wood and products of wood and cork; Paper: paper products and printing; Petroleum: coke and refined petroleum products; Chemicals: chemicals and pharmaceutical products; Plastic: rubber and plastic products; Minerals: other nonmetallic mineral products; Basic metals; Metal products: fabricated metal products; Computer: computer, electronic and optical products; Electrical: electrical equipment; Machinery nec: machinery and equipment, nec; Motor vehicles: motor vehicles, trailers, and semi-trailers; Other transport: other transport equipment; Other: other manufacturing, repair, and installation of machinery and equipment.

Figure 5 shows the sectoral contribution to aggregate CO_2_ emission changes generated by trade changes for RCEP members. The exports of electrical equipment contribute the most to China (14.5%), the ASEAN countries (12.7%), Japan (18.7%), and South Korea (23.7%). The other two large contributors are machinery and equipment (second largest for China and the ASEAN countries, third-largest for Japan and South Korea) and computer, electronic, and optical products (second for South Korea, third for China, and fifth for Japan). The motor vehicle industry makes the second-largest contribution (17.8%) to Japan, but its contribution to other countries is relatively small, indicating Japan’s strong comparative advantage in this industry. For Australia and New Zealand, the increased trade generates a very small increase in emissions. The major contributors are mining for Australia and agriculture for New Zealand.

The finding that RTAs increase participants’ economic welfare at the cost of environmental burdens can be explained by the fact that in real economic interactions, what, how much, and with whom to trade are still determined based on economic profitability rather than environmental considerations. In a Ricardian world, a country exports more products in which it has a comparative advantage in terms of production. The advantage is defined as using fewer of the resources under consideration. Traditionally, labor was such a resource, and additional trade generated from lower trade costs led to increased welfare and emissions in each trading country. Alternatively, if we consider environmental aspects, the story changes. For instance, if we define the advantage as generating fewer emissions in producing a certain product^37^, increased trade in this product will reduce emissions in both countries.

### Global value chain participation and emission intensity

The main channels through which a country’s emission is determined are the scale effects, the sectoral composition effects, and the technique (intensity) effects^30^. The results of emission burdens presented above focus on the first two channels holding the last channel constant. Next, we examine how trade liberalization following RCEP may affect the emission intensity and whether such effects lessen or intensify the emission burdens driven by the scale effects and the sectoral composition effects. Specifically, we examine the effects of global value chain (GVC) participation on emission intensity. GVC participation indicators measure to what extent countries/industries/firms are involved in globally fragmented production. GVC participation may affect emission intensity via pollution outsourcing arising from imports of intermediate inputs, intensified competition or knowledge spillover which encourages process innovation and technological upgrading, and other channels^29^. As the deepened GVC participation can be considerably attributed to the reduction of trade costs in the past decades^38^, we use GVC participation as a proxy of trade liberalization to examine its effects on emission intensity. We distinguish between forward and backward GVC participation because they reflect two different ways of participating in GVCs. Therefore, they may have different effects on the emission intensity of developed countries and developing countries. The forward participation reflects a country’s supplying intermediates to other countries for further production, while the backward GVC participation measures to what extent the country imports intermediate inputs to produce its products^39^.

In Supplementary Note 2, we provide in detail how we measure forward and backward GVC participation using the ICIO table and how we capture the effects of GVC participation on emission intensity using panel regression techniques. Table 2 presents the results. We observe that the effects of forward and backward participation on developed countries and developing countries are different. Forward participation has significant reducing effects on the emission intensity for developed countries, while it has no consistent significant effects for developing countries. A possible explanation for this observation could be their difference in forward GVC participation. For developed countries, forward integration into GVCs typically means outsourcing parts of relatively dirty production activities. This process helps to lower its emission intensity. However, for developing countries, forward participation in GVCs tends to be providing (cheap) labor and raw materials and specializing in emission-intensive production^40^. This process may generate economic gains and increase emission levels but does not necessarily alter emission intensity in developing countries.

**Table 2:** GVC participation and emission intensity.

Emission intensity		Emission intensity	
Independent variable		Independent variable	
Forward	0.04 (0.07)	Backward	−0.03 (0.02)
Forward*d_*r*_	−0.07 (0.07)	Backward *d_*r*_	0.09 (0.08)
Forward (−1)	0.06 (0.05)	Backward (−1)	−0.08 (0.05)
Forward (−1)*d_*r*_	−0.11^**^(0.06)	Backward (−1)*d_*r*_	0.07 (0.05)
Forward (−3)	0.12^*^ (0.03)	Backward (−3)	−0.11^*^ (0.07)
Forward (−3)*d_*r*_	−0.19^***^(0.04)	Backward (−3)*d_*r*_	0.06^*^ (0.03)
Forward (−5)	0.04^*^ (0.02)	Backward (−5)	−0.16^*^ (0.12)
Forward (−5)*d_*r*_	−0.12^**^ (0.03)	Backward (−5)*d_*r*_	0.05^**^ (0.02)
Forward (−7)	0.01 (0.04)	Backward (−7)	−0.21^***^(0.10)
Forward (−7)*d_*r*_	−0.09^**^ (0.04)	Backward (−7)*d_*r*_	0.10^**^ (0.04)
Forward (−9)	0.02 (0.03)	Backward (−9)	−0.28^***^(0.13)
Forward (−9)*d_*r*_	−0.13^**^ (0.03)	Backward (−9)*d_*r*_	0.15^**^ (0.08)
Country–industry fixed effect	Yes	Country–industry fixed effect	Yes
Country–year fixed effect	Yes	Country–year fixed effect	Yes
Industry–year fixed effect	Yes	Industry–year fixed effect	Yes

Note: ***P < 0.01.

**P < 0.05.

*P < 0.1.

Standard errors are reported in parentheses.

The variables “forward” and “backward” refer to as “forward participation” and “backward participation”, respectively (Supplementary Note 2). Variables of GVC participation and emission intensity are in natural logarithms. The adjusted *R*^2^ for all regressions is larger than 0.65. The sample covers 36 sectors for 64 economies over 11 years (2005–2015). *d*_*r*_ denotes the development stage. Developed countries (*d*_*r*_ = 1) refer to as high-income economies and developing countries (*d*_*r*_ = 0) refer to as middle- and low-income economies, which are classified according to World Bank’s income classification in 2005. The notations (-1), (-3), and the rest denote a lag of 1-year, 3-year, etc.

Backward GVC participation has no significant effects until an extended time period (about 3–5 years). The effects do not materialize in the short run. Deeper backward participation indicates that a country increases its imports of intermediate inputs to process into products for domestic consumption and/or export. It does not suggest that the country will necessarily substitute its domestic production with imports. This explains that we do not observe a significant negative effect on emission intensity contemporaneously. In the medium and long run, we observe that increased backward participation reduces emission intensity for both developed and developing countries, and it reduces more of the emission intensity for developing countries than for developed countries. It increases a country’s foreign market access to cleaner intermediate inputs, creating knowledge spillovers, stimulating upgrading, and thus reducing the emission intensity. However, such upgrading-by-doing effects materialize in the long run rather than contemporaneously. The results also suggest that backward participation provides more upgrading opportunities for a developing country. This finding is consistent with the convergence theory that learning-by-doing enables lagging countries to catch up with the leaders of emission efficiency in the longer run.

Forward and backward GVC participation focuses on the effects of trade in intermediate inputs on emission intensity. We also examine the effects of trade in final goods on emission level and emission intensity. We find that imports of final goods can substitute domestic production and thus reduce domestic emissions. However, we find no robust significant effect of trade in final goods on emission intensity, which attests that trade in final goods alters a country’s emission level but does not necessarily change the emission intensity.

Comprehensively considering the effects of GVC participation and trade in final goods, we conclude that trade liberalization reduces the emission intensity of developed countries to a larger extent. The effects of GVC participation on developing countries materialize mainly via backward participation in the medium and long run. Therefore, we conclude that the intensity effects of deeper GVC participation following RCEP can lessen the emission burdens that we present in the section above for developed members such as Japan and South Korea in a relatively short run. The intensity effects can also lessen the emission burdens for developing countries. However, it takes longer, and the intensity effects are not strong enough to completely offset the burdens. To be specific, our estimations indicates that China’s emission intensity can potentially decline by 0–1.8% due to the deeper GVC participation. This reduction in emission is not large enough to offset China’s burdens, recalling the results above that RCEP increases China’s emissions by 2.7% and 4.0%, respectively, in the 5th and 10th year after it enters into force. A similar situation applies to most developing ASEAN economies.

## Discussion

The world trade system has been seriously undermined due to huge shocks, such as the COVID-19 global pandemic, the United States–China trade conflict, and Brexit, which to some extent have stimulated the formation of more RTAs. In this paper, we estimate the economic gains and the corresponding carbon-emission burdens of the RCEP. The results show that RCEP tariff elimination will substantially reduce intraregional trade costs and product prices, increase the comparative advantage of regional products, and ultimately improve the welfare of all member countries. Meanwhile, we point out that policymakers should pay attention to the environmental impacts of the RCEP since our results indicate non-negligible increases in potential CO_2_ emissions caused by the RCEP. However, we emphasize that anti-globalization is far from a possible strategy for global emission mitigation. Although de-globalization could reduce international trade and the corresponding embodied carbon emissions in the short term^41^, it harms the economic welfare of all countries and threatens international efforts to fight climate change in the longer run. Our regression results show that deeper GVC participation reduces the emission intensities of the participating countries. Returning to autarky cuts off developing countries’ opportunities to participate in GVCs and then upgrade their emission technologies through learning-by-doing. In addition, anti-globalization will hinder international cooperation that aims to mitigate global emissions^42^.

Instead, we emphasize that technological advancements in reducing pollutant intensity are urgently required in more developing countries. We find that the carbon-emission intensities (CO_2_ emissions per unit of output) in all member countries should decline to offset the extra emissions caused by the RCEP, particularly for the developing member countries. Our calculations based on data for 2015 show that the emission intensities of China (2.83 times) and most ASEAN economies, such as Vietnam (2.25 times), Malaysia (2.11 times), and Thailand (2.04 times), were more than twice that of Japan. Our regression results based on historical data show that the technological spillover effects of trade integration are not strong enough to offset the emission burdens for developing countries. Therefore, on the one hand, strengthening regional and international coordination between developed RCEP members and developing members are very necessary to accelerate the diffusion of cleaner production technologies to the developing members when implementing the trade agreement. This process will help accelerate improvements in emission performance in developing countries. On the other hand, developing nations should accelerate efforts to reduce their emission intensity gap with developed nations. For example, as the world’s factory and the largest emitter of CO_2_, in 2021, China pledged to achieve carbon neutrality before 2060, which is largely consistent with the 1.5 °C warming limit^43^. The country is making greater efforts and taking a series of measures to achieve this challenging goal.

Since all the RCEP countries have committed to the Paris Agreement and the SDG agenda of the United Nations, these nations are highly motivated to ensure economic development and environmental sustainability in tandem^12^. The majority of the RCEP members should mitigate their fossil fuel dependencies and improve the share of renewable energy in their energy consumption basket. It is suggested that research and development be strengthened to develop renewable energy and enhance technological innovations to reduce pollutant emission intensity further. Investments (e.g., via carbon tax, green bonds, or other relevant financial tools) should be directed at relatively greener production and consumption activities across these nations.

Our findings also suggest that more effective climate policies for international trade should be designed and implemented as we find that some members will emit increasing CO_2_ for other countries. Some often discussed policies include levying carbon tax on international trade and setting an international carbon price floor with border tax adjustments^44^. We stress that the premise of such policies is a robust and fair accounting system to assign responsibility for internationally traded emissions. Currently, the production-based accounting (PBA) system is in practice widely adopted to assign responsibilities for global environmental problems to individual countries, but it ignores potential carbon leakages through international trade. Consumption-based accounting (CBA) includes the emissions that are emitted at home or in a foreign country but which are embodied in the final products that are consumed at home. CBA redistributes the emissions from PBA, but it still has its problems^37^. Several researchers proposed further refinements in CBA for assigning the responsibilities for global emissions^45–52^. Despite these efforts in academia, national and global climate policies have not adopted such adjusted accounting systems so far. Therefore, we call for the idea of governments and researchers working together to design robust and fair accounting tools, develop and implement effective global and regional climate policies, and share the responsibility of global emission mitigation^53–56^.

This study has potential extensions that are worthy of pursuit. First, we did not measure other potential environmental impacts, of which the most important include air pollutants (e.g., fine particulate matter, PM_2.5_) associated with fuel burning. Second, we currently measured economic welfare due to tariff reduction following RCEP. We did not incorporate endogenous environmental regulations into the equilibrium model. Therefore, the negative welfare effects caused by pollutant emissions are not included in the welfare. Future studies can extend the model by incorporating environmental regulation into the production function and household utility function^31^. The extended model can be used to conduct policy analysis by investigating potential environmental regulations that align with economic development goals and optimize the economic and environmental welfares of RCEP members. For example, to explore optimal emission tax levels that maximize the welfares of a specific RCEP member after the agreement enters into force.

Third, we did not consider the influence mechanism by which the barriers in services trade and investment in the RCEP region will also decrease under the agreement. Both international trade and cross-border investment can influence a country’s economic welfare and environmental issues. Although global flows of FDI shrank in recent years^57,58^ and fell sharply by one third^59^ in 2020 due to the COVID-19 pandemic, FDI among RCEP members will likely continuously increase after the RCEP enters into force. Increasing FDI may facilitate relocating some climate-unfriendly industries or production activities from industrialized RCEP members to developing countries^24,25^. FDI may also bring cleaner technology to developing members, which would help reduce the emission intensities. As a result, FDI may cause multidimensional environmental influence on RCEP members. Future studies are expected to provide quantitative analyses of the effects of qualitative cross-border investment rules in the RCEP Agreement on the volume and direction of FDI flows. More in-depth analyses are also expected to allocate the carbon footprints of FDI flows and explore sharing environmental responsibility between FDI home and host countries.

## Methods

### Quantifying the economic effects

This section outlines the model^13^ that we employ to quantify the effects of RCEP tariff reductions on trade and welfare. The world consists of *n* countries, and there are *m* sectors in each country. Countries are denoted by *s* and *r* and sectors by *i* and *j*. The households in country *r* derive utility from consuming final products ${C}_{r}^{j}$, and ${\alpha }_{r}^{j}$ is the corresponding sectoral consumption weight. The function is Cobb–Douglas and given by

1 $$u\left({C}_{r}\right)=\mathop {\prod }\limits_{j=1}^{m}{{C}_{r}^{j}}^{{\alpha }_{r}^{j}},{{{{{\rm{where}}}}}}\,\mathop{\sum }\limits_{j=1}^{m}{\alpha }_{r}^{j}=1$$

There is a continuum of intermediate products *ω*^*j*^ produced in sector *j*. Primary inputs (labor) and a bundle of intermediate inputs from all sectors are used for the production of *ω*^*j*^ in country *r*. The production technology is

$${q}_{r}^{j}\left({\omega }^{j}\right)={z}_{r}^{j}\left({\omega }^{j}\right){\left[{{lab}}_{r}^{j}\left({\omega }^{j}\right)\right]}^{{\gamma }_{r}^{j}}\mathop {\prod }\limits_{i=1}^{m}{\left[{{int}}_{r}^{i,j}\left({\omega }^{j}\right)\right]}^{{\gamma }_{r}^{i,j}},$$

where ${z}_{r}^{j}$(*ω*^*j*^) denotes the efficiency in producing *ω*^*j*^ in country *r*. ${{lab}}_{r}^{j}$(*ω*^*j*^) is labor and ${{int}}_{r}^{i,j}$(*ω*^*j*^) are the intermediate inputs from sector *i* required in the production of *ω*^*j*^. ${\gamma }_{r}^{j}$ denotes the share of value-added in production output, and ${\gamma }_{r}^{i,j}$ denotes the share of products from sector *i* used as intermediate inputs in the production of *ω*^*j*^, with ${\sum }_{i=1}^{m}{\gamma }_{r}^{i,j}+{\gamma }_{r}^{j}=1$. The cost of an input bundle is given by

2 $${c}_{r}^{j}={B}_{r}^{j}{{w}_{r}}^{{\gamma }_{r}^{j}}\mathop {\prod }\limits_{i=1}^{m}{{P}_{r}^{i}}^{{\gamma }_{r}^{i,j}},$$

where ${w}_{r}$ denotes the wage rate, ${P}_{r}^{i}$ gives the price of intermediate inputs from sector *i*, and ${B}_{n}^{j}$ is a constant. ${c}_{r}^{j}$ clearly incorporates all the intersectoral linkages which can be obtained from input–output tables.

In an open economy, producers minimize their production costs and purchase intermediate products from suppliers across countries. However, trade is costly. We denote ${k}_{{rs}}^{j}$ as the bilateral trade cost for country *r*’s imports of sector *j* products shipped from country *s*. It consists of an ad valorem tariff (${\tau }_{{rs}}^{j}$) and iceberg trade cost (${d}_{{rs}}^{j}$), ${k}_{{rs}}^{j}=(1+{\tau }_{{rs}}^{j}){d}_{{rs}}^{j}$. Using Eaton and Kortum’s^60^ representation of technologies which allows production efficiency to distribute Fréchet, we can derive the price of the intermediate product as

3 $${P}_{r}^{j}={G}^{j}{\left[\mathop{\sum }\limits_{s=1}^{n}{\lambda }_{s}^{j}{\left({c}_{s}^{j}{k}_{{rs}}^{j}\right)}^{-{\theta }^{j}}\right]}^{-1/{\theta }^{j}},$$

where ${G}^{j}$ is a constant, ${\lambda }_{s}^{j}$ reflects absolute advantage as a higher value indicates more likely a draw of high efficiency, and ${\theta }^{j}$ captures comparative advantage as a lower value indicates a higher dispersion of efficiency. ${\lambda }_{s}^{j}$ and ${\theta }^{j}$ reflect Ricardian trade^61^.

The properties of Fréchet distribution further enable us to derive the bilateral trade share as

4 $${\pi }_{{rs}}^{j}=\frac{{\lambda }_{s}^{j}{\left[{c}_{s}^{j}{k}_{{rs}}^{j}\right]}^{-{\theta }^{j}}}{\mathop{\sum }\limits_{h=1}^{n}{\lambda }_{h}^{j}{\left[{c}_{h}^{j}{k}_{{rh}}^{j}\right]}^{-{\theta }^{j}}}.$$

As shown, any changes in tariffs (${\tau }_{{rs}}^{j}$) can affect trade costs (${k}_{{rs}}^{j}$) and thus directly affect trade shares. Equations (2) and (3) show that changes in tariffs also affect the cost of input bundle (${c}_{s}^{j}$) and thus have an indirect effect on trade.

The total expenditure on the products of sector *j* in country *r* is the summation of firms’ expenditures on intermediate products and households’ expenditures on final products. It is given by

5 $${X}_{r}^{j}=\mathop{\sum }\limits_{i=1}^{m}{\gamma }_{r}^{j,i}\mathop{\sum }\limits_{s=1}^{n}{X}_{s}^{i}\frac{{\pi }_{{sr}}^{i}}{1+{\tau }_{{sr}}^{i}}+{\alpha }_{r}^{j}{I}_{r},$$

where

6 $${I}_{r}={{w}_{r}L}_{r}+{R}_{r}+{D}_{r}$$

represents the total household income in country *r*, i.e., the sum of labor income (${{w}_{r}L}_{r}$), tariff revenues (*R*_*r*_) and trade deficits (*D*_*r*_). In particular, ${R}_{n}={\sum }_{j=1}^{m}{\sum }_{s=1}^{n}{\tau }_{{rs}}^{j}{M}_{{rs}}^{j}$, where ${M}_{{rs}}^{j}={X}_{r}^{j}\frac{{\pi }_{{rs}}^{j}}{1+{\tau }_{{rs}}^{j}}$ is country *r*’s import of sector *j* products from country *s*. The trade deficit of a country is the summation of sectoral deficits, ${D}_{r}={\sum }_{j=1}^{m}{D}_{r}^{j}$, and sectoral deficit is given by ${D}_{r}^{j}={\sum }_{s=1}^{n}{M}_{{rs}}^{j}-{\sum }_{s=1}^{n}{E}_{{rs}}^{j}$, where ${E}_{{rs}}^{j}={X}_{s}^{j}\frac{{\pi }_{{sr}}^{j}}{1+{\tau }_{{sr}}^{j}}$.

The next step is to solve for changes in wages and prices given that the tariff structure *τ* is changed to *τ*′. Instead of solving for two equilibria under *τ* and *τ*′, Caliendo and Parro^13^ propose solving for an equilibrium in relative changes so that it is not necessary to estimate some parameters that are difficult to identify. Let a variable with a circumflex “$\hat{x}$” denote its relative change. The equilibrium in relative changes satisfies the following conditions:

7 $${\hat{k}}_{{rs}}^{j}=(1+{\tau }_{{rs}}^{{j}^{{\prime} }})/(1+{\tau }_{{rs}}^{j})$$

8 $${\hat{c}}_{r}^{j}={{\hat{w}}_{r}}^{{\gamma }_{r}^{j}}\mathop{\prod }\limits_{i=1}^{m}{\hat{P}}_{r}^{i{\gamma }_{r}^{i,j}}$$

9 $${\hat{P}}_{r}^{j}={\left[\mathop{\sum }\limits_{s=1}^{n}{\pi }_{{rs}}^{j}{\left({\hat{c}}_{s}^{j}{\hat{k}}_{{rs}}^{j}\right)}^{-{\theta }^{j}}\right]}^{-1/{\theta }^{j}}$$

10 $${\hat{\pi }}_{{rs}}^{j}={\left[\frac{{\hat{c}}_{s}^{j}{\hat{k}}_{{rs}}^{j}}{{\hat{P}}_{r}^{j}}\right]}^{{-\theta }^{j}}$$

11 $${X}_{r}^{{j}^{{\prime} }}=\mathop{\sum }\limits_{i=1}^{m}{\gamma }_{r}^{j,i}\mathop{\sum }\limits_{s=1}^{n}{X}_{s}^{{i}^{{\prime} }}\frac{{\pi }_{{sr}}^{{i}^{{\prime} }}}{1+{\tau }_{{sr}}^{{i}^{{\prime} }}}+{\alpha }_{r}^{j}{I}_{r}^{{\prime} }$$

12 $$\mathop{\sum }\limits_{j=1}^{m}\mathop{\sum }\limits_{s=1}^{n}{X}_{r}^{{j}^{{\prime} }}\frac{{\pi }_{{rs}}^{{j}^{{\prime} }}}{1+{\tau }_{{rs}}^{{j}^{{\prime} }}}-{D}_{r}^{{\prime} }=\mathop{\sum }\limits_{j=1}^{m}\mathop{\sum }\limits_{s=1}^{n}{X}_{s}^{{j}^{{\prime} }}\frac{{\pi }_{{sr}}^{{j}^{{\prime} }}}{1+{\tau }_{{sr}}^{{j}^{{\prime} }}}$$

13 $${I}_{r}^{{\prime} }={{\hat{w}}_{r}{w}_{r}L}_{r}+{R}_{r}^{{\prime} }+{D}_{r}^{{\prime} }.$$

Introducing into the model the changes in tariff structure, we can solve for changes in (total and bilateral) trade flows, real wages (${w}_{r}/{P}_{r}$), welfare (${I}_{r}/{P}_{r}$), and production output for each country. The change in welfare can be decomposed into volume of trade effect and terms of trade effect. The welfare change can also be calculated at both the bilateral and sectoral levels. We can calculate the change in volume of trade and terms of trade between country *s* and *r*, and the change in a specific sector *j* of country *r*.

### Accounting for the carbon-emission changes

The RCEP tariff reductions lead to changes in multilateral trade flows resulting in trade-related carbon emissions changes. We adopt the environmentally extended ICIO model (see Supplementary Table 2 for the stylized table^62^) to account for the carbon-emission changes. Denote the following as the flows of final products among different countries:And,is the global direct input–output coefficient matrix. Its typical element ${a}_{{ij}}^{{sr}}$ provides the intermediate input from sector $i(=1,\cdots ,m)$ in country $s(=1,\cdots ,n)$ used by sector $j(=1,\cdots ,m)$ in country $r(=1,\cdots ,n)$ for producing one unit of output. ${{{{{{\bf{A}}}}}}}^{{rr}}$ and ${{{{{{\bf{f}}}}}}}^{{rr}}$ provide intra-country flows of intermediate products and final products. ${{{{{{\bf{A}}}}}}}^{{sr}}$ and ${{{{{{\bf{f}}}}}}}^{{sr}}(s\,\ne\, r)$ represent trade in intermediate products and trade in final products, respectively. According to the standard input–output model^31^, the gross output vector **y** is

14 $${{{{{\bf{y}}}}}}={\left({{{{{\bf{I}}}}}}-{{{{{\bf{A}}}}}}\right)}^{-1}{{{{{\bf{Fu}}}}}},$$

where **I** is an (*nm* × *nm*) identity matrix, and **u** is a summation vector of appropriate length with all elements being ones.

Let **w** be the CO_2_ emission coefficient vector, the elements of which provide the emissions per unit of output. Then, the CO_2_ emission vector **e** can be written as

15 $${{{{{\bf{eu}}}}}}={{{{{{\bf{w}}}}}}\left({{{{{\bf{I}}}}}}-{{{{{\bf{A}}}}}}\right)}^{-1}{{{{{\bf{Fu}}}}}}.$$

The left side of Eq. (15) equals the summation of CO_2_ emissions in all sectors in the world, which equals global CO_2_ emissions. This equation can be adapted to allow us to calculate the CO_2_ emissions (${{{{{{\rm{e}}}}}}}^{r}$) in a specific country $r$. This can be obtained by replacing the vector ${{{{{\bf{w}}}}}}$ in Eq. (15) with a vector ${{{{{{\bf{w}}}}}}}^{r}$. The new vector has equal length, but only the CO_2_ emission coefficients for the sectors in country $r$ are retained while all other elements are set as zeros. This yields

16 $${{{{{{\rm{e}}}}}}}^{r}={{{{{{{\bf{w}}}}}}}^{r}\left({{{{{\bf{I}}}}}}-{{{{{\bf{A}}}}}}\right)}^{-1}{{{{{\bf{Fu}}}}}}$$

Equation (16) allows us to calculate, for example, the part of Thailand’s CO_2_ emissions that are generated by the exports of final products from Japan to final users in China. The production processes for trade between Japan and China may consume intermediate products from Thailand, of which the production emits CO_2_ in Thailand. Using the global Leontief inverse, we can take fully into account these indirect effects in Eq. (16).

To calculate the CO_2_ emission changes in country $r$, we compare two situations. The first is the actual situation, and the second is the case in which multilateral trade flows are changed due to RCEP tariff reductions. Moving forward from Eq. (16), we develop the following equation:

17 $${\triangle {{{{{\rm{e}}}}}}}^{r}={{{{{{{\bf{w}}}}}}}^{r}\left({{{{{\bf{I}}}}}}-\widetilde{{{{{{\bf{A}}}}}}}\right)}^{-1}\widetilde{{{{{{\bf{F}}}}}}}{{{{{\bf{u}}}}}}-{{{{{{{\bf{w}}}}}}}^{r}\left({{{{{\bf{I}}}}}}-{{{{{\bf{A}}}}}}\right)}^{-1}{{{{{\bf{Fu}}}}}},$$

whereEquation (17) enables us to consider the changes in both trade in final products and trade in intermediate products. The changes in bilateral trade flows can be obtained at the sectoral level after solving the equilibrium model described above.

## Supplementary information

Supplementary information

## Data Availability

National and ICIO tables are from the most recent OECD Input–Output Database^63^ (2018 edition, https://stats.oecd.org/), and bilateral trade flows are obtained from the United Nations Commodity Trade (U.N. Comtrade) database (https://comtrade.un.org/data/). Bilateral tariff data are from World Integrated Trade Solution (WITS) software (https://wits.worldbank.org/). The committed tariff reductions among RCEP parties come from the Schedule of Tariff Commitments in the RCEP Agreement. The schedule provides detailed data for each RCEP party’s commitments to tariff reduction for each year after the date of the entry into force of the RCEP Agreement. International Energy Agency (https://www.iea.org/data-and-statistics) provides sectoral CO_2_ emissions data^11^, and the most recent available data are for 2018. We include the maximum number of economies, conditional on obtaining reliable data. We ultimately obtain 60 economies and a constructed RoW with 36 sectors in each economy. Trade and tariff data are for 2019, and we employ the most recent available input–output tables for 2015, assuming that input–output coefficients in 2019 are not much different from those in 2015. Supplementary Note 1 provides more detailed descriptions of all data used in our evaluation. Supplementary Tables provide additional results. All datasets generated in this study are available upon reasonable request.
