# Supplementary material for: Regional trade agreement burdens global carbon emissions mitigation
Source: Nat Commun. 2022 Jan 20;13:408. doi: 10.1038/s41467-022-28004-5 (PMC8776788; doi:10.1038/s41467-022-28004-5)
Supplement: Supplementary file 1 — Supplementary information [file 41467_2022_28004_MOESM1_ESM.docx]

**Supplementary Information for**

**Regional trade agreement burdens global carbon emissions mitigation**

Kailan Tian^1^, Yu Zhang^1^, Yuze Li^2^, Xi Ming^3^, Shangrong Jiang^3^, Hongbo Duan^3*^, Cuihong Yang^1,3*^, Shouyang Wang^1,3*^

1. NCMIS, MADIS, Academy of Mathematics and Systems Science, Chinese Academy of Sciences, Beijing 100190, China

2. Questrom School of Business, Boston University, Boston 02215, USA

3. School of Economics and Management, University of Chinese Academy of Sciences, Beijing 100190, China

**Corresponding authors: H.D. (**[**hbduan@ucas.ac.cn**](mailto:hbduan@ucas.ac.cn)**), C.Y. (**[**chyang@iss.ac.cn**](mailto:chyang@iss.ac.cn)**), S.W. (**[**sywang@amss.ac.cn**](mailto:sywang@amss.ac.cn)**)**

**This file includes:**

Supplementary notes (Note 1 and Note 2)

Supplementary figures (Figure 1)

Supplementary tables (Table 1-12)

**Supplementary note 1. Data sources and description**

We include 60 economies and a constructed rest of the world in our analysis. The list of economies is: Argentina, Brazil, Canada, Switzerland, Chile, Colombia, Costa Rica, EU (28 members, including the United Kingdom), India, Iceland, Israel, Morocco, Mexico, Norway, Russia, Saudi Arabia, Turkey, United States, South Africa, Australia, Brunei, mainland China, Hong Kong, China, Indonesia, Japan, Cambodia, Korea, Malaysia, New Zealand, Philippines, Singapore, Thailand, Vietnam. There are 36 sectors (20 tradable and 16 non-tradable) in each economy. The list of sectors is reported in Table A1.

We use four types of data: input-output (IO) tables, bilateral trade flows, bilateral tariff data, sectoral CO_2_ emission data.

**Input-output tables**. We obtain national input-output tables from the most updated 2018 edition of OECD Input-Output Database. The database provides annual IO tables for 64 economies for the period 2005-2015 with 36 ISIC Rev. 4 industries in each economy (Table A1). We calculate the input-output coefficients using the most recent available IO tables for the year 2015. The OECD database also provides annual inter-country IO (ICIO) tables (see Table A2 for the stylized table) for the period 2005-2015, enabling us to calculate emissions embodied in trade. The ICIO tables are global because they include 64 economies and provide an estimate of the economic structure in the “Rest of the World”. We employ the ICIO table for the year 2015.

**Bilateral trade data**. We employ bilateral trade flows from the United Nations Commodity Trade (UN Comtrade) database. The traded goods are defined using the Harmonized Commodity Description and Coding System (HS) at the 6-digital level. We concord the goods at HS-6 level to the 20 tradable industries (2-digit ISIC Rev. 4) using the United Nations concordance table. We use the bilateral trade data for our sampled economies for the year 2019.

**Emission data**. Sectoral CO_2_ emissions data come from the International Energy Agency, and the most recent available data are for the year 2018. In the calculation, we use the emission intensities (emissions per unit of output) of all economies for 2015 to calculate the absolute changes in the amount of CO_2_ emissions as the sectoral outputs for the sampled countries are not available for 2018. However, we calculate the change rates as presented in Fig.3 using the emissions in 2018 as the base values.

**Bilateral tariff data**. The bilateral tariff data before the entry of RCEP into force are obtained from the World Integrated Trade Solution (WITS) software, which is originally from the United Nations Statistical Division-Trade Analysis and Information System (UNCTAD-TRAINS). We employ effective applied rates for 2019 as they are the actual tariff applied, which already took into account all existing trade agreements (except the RCEP) between the countries. The original tariff data are at HS-8 level, and we concord them to ISIC Rev. 4 industries using the simple averages. We also checked the results by using weighted averages and the results are robust.

The tariff structure after the entry of RCEP into force is obtained from the Schedule of Tariff Commitments in the RCEP agreement. The Schedule provides detailed data of each RCEP party’s commitments of tariff reduction in each year since the date of entry into force of the RCEP Agreement. The base tariffs in the Schedule are the most-favoured-nation (MFN) tariffs for the year 2014. Therefore, the MFN tariffs in the Schedule after RCEP enters into force in some year could be higher than the effective applied rates for 2019. We thus set the minimum values of the two tariff structures as the applied tariff structure after RCEP enters into force in that year.

**Schedule of tariff reduction in RCEP agreement**.

This part we present the simple average import tariff imposed by RCEP members on other members after the RCEP agreement enters into force (see Supplementary Figure 1).

**Supplementary note 2. Measuring the effects of global value chain participation on emission intensity**

**Forward and backward GVC participation indicators**

GVC participation indicators measure to what extent countries/industries/firms are involved in globally fragmented production. We measure GVC participation from backward linkages and forward linkages, reflecting the two different ways of participating GVCs. The forward participation reflects a country’s supplying intermediates to other countries for further production, while the backward GVC participation measures to what extent the country imports intermediate inputs to produce its products.

We follow the work by Tian et al. (2021)^1^ and Wang et al. (2017)^2^ and construct forward and backward GVC participation indicators by decomposing value-added and final goods in an ICIO model (see Supplementary Table 2). There are $n$ countries and $m$ sectors (or industries) in each country. In compact form, the static ICIO system can be written as $\mathbf{y=Ay+f}$ and the solution is

$\mathbf{y=}\left( \mathbf{I-A} \right)^{-1}\mathbf{f=Bf}$, (A1)

where the vector $\mathbf{y}$ gives all country-sectors’ gross outputs (inputs), $\mathbf{A}$ the global input-output matrix, and $\mathbf{f}$ the vector of final demand. $\mathbf{I}$ is an identity matrix. $\boldsymbol{B\equiv}\left( \mathbf{I-A} \right)^{-1}$ is the global Leontief inverse.

Both the intermediate input matrix and final demand vector can be decomposed into two parts. It yields

$\mathbf{y}=\mathbf{Ay+f}=\mathbf{A}^{\mathbf{D}}\mathbf{y+}\mathbf{f}^{\mathbf{D}}+\mathbf{A}^{\mathbf{F}}\mathbf{y+}\mathbf{f}^{\mathbf{F}}$, (A2)

where $\mathbf{A}^{\mathbf{D}}=\left[ \begin{matrix} \begin{matrix} \mathbf{A}^{11} \\ \boldsymbol{\vdots} \end{matrix} & \begin{matrix} \boldsymbol{\cdots} & \mathbf{O} & \boldsymbol{\cdots} \\ \boldsymbol{\ddots} & \boldsymbol{\vdots} & \boldsymbol{⋰} \end{matrix} & \begin{matrix} \mathbf{O} \\ \boldsymbol{\vdots} \end{matrix} \\ \mathbf{O} & \begin{matrix} \boldsymbol{\cdots} & \mathbf{A}^{rr} & \boldsymbol{\cdots} \end{matrix} & \mathbf{O} \\ \begin{matrix} \boldsymbol{\vdots} \\ \mathbf{O} \end{matrix} & \begin{matrix} ⋰ & \boldsymbol{\vdots} & \boldsymbol{\ddots} \\ \boldsymbol{\cdots} & \mathbf{O} & \boldsymbol{\cdots} \end{matrix} & \begin{matrix} \boldsymbol{\vdots} \\ \mathbf{A}^{nn} \end{matrix} \end{matrix} \right]$ is a diagonal block matrix of domestic input coefficients with $\mathbf{O}$ standing for a $m\times m$ matrix filled with zeros. $\mathbf{A}^{\mathbf{F}}$ is an off-diagonal matrix of imported input coefficients, and $\mathbf{A}^{\mathbf{F}}=\mathbf{A}-\mathbf{A}^{\mathbf{D}}$. $\mathbf{f}^{\mathbf{D}}=\left( \begin{matrix} \begin{matrix} \mathbf{f}^{11} \\ \boldsymbol{\vdots} \end{matrix} \\ \mathbf{f}^{rr} \\ \begin{matrix} \boldsymbol{\vdots} \\ \mathbf{f}^{nn} \end{matrix} \end{matrix} \right)$ is a vector of final demand for domestic final products, and ${\mathbf{f}^{\mathbf{F}}\mathbf{=f-f}}^{\mathbf{D}}$ is a vector of final demand for foreign final products. Rearranging equation (A2) yields

$\mathbf{y}=\left( \mathbf{I}-\mathbf{A}^{\mathbf{D}} \right)^{-1}\mathbf{f}^{\mathbf{D}}+\left( \mathbf{I}-\mathbf{A}^{\mathbf{D}} \right)^{-1}\left( \mathbf{A}^{\mathbf{F}}\mathbf{y}+\mathbf{f}^{\mathbf{F}} \right)=\mathbf{L}\mathbf{f}^{\mathbf{D}}+\mathbf{L}\mathbf{f}^{\mathbf{F}}+\mathbf{L}\mathbf{A}^{\mathbf{F}}\mathbf{Bf}$ , (A3)

where ${\mathbf{L}=\left( \mathbf{I}-\mathbf{A}^{\mathbf{D}} \right)}^{-1}$ is a diagonal block that denotes the domestic Leontief inverse.

Let $(\mathbf{v}^{s})'=(\mathbf{w}^{s})'\left( {\hat{\mathbf{y}}}^{s} \right)^{-1}$ be the row vector which gives the value-added coefficients in country $s$. Combining this with equation (A3), we can decompose the sectoral value-added of country $s$ as

$\mathbf{w}^{s}\boldsymbol{=}{\hat{\mathbf{v}}}^{s}\mathbf{y}^{s}\boldsymbol{=}{\hat{\mathbf{v}}}^{s}\mathbf{L}^{ss}\mathbf{f}^{ss}\boldsymbol{+}{\hat{\mathbf{v}}}^{s}\mathbf{L}^{ss}\sum_{r\neq s}^{n} \mathbf{f}^{sr}\boldsymbol{+}{{\hat{\mathbf{v}}}^{s}\mathbf{L}}^{ss}\sum_{r\neq s}^{n} \mathbf{A}^{sr}\sum_{u}^{n} \mathbf{B}^{ru}\boldsymbol{(}\sum_{g}^{n} \mathbf{f}^{ug}\boldsymbol{)}$. (A4)

Equation (A4) gives the distribution of value-added created in one country-sector. The first term is domestic value-added (DVA) in the production of final products for domestic demand without border-crossing production activities. The second term is DVA in the production of final products exports, which does not involve any border-crossing production activities either. This part of DVA crosses national borders once, but only for final consumption. The third term is DVA in the production of intermediate exports. It is DVA that relates to production activities outside the source country and it denotes the source country’s contribution to global production.

The forward GVC participation indicator ($\mathbf{fgvc}$) is the DVA embodied in the exports of intermediate products as a share of the country-sector’s total value-added, namely

$\mathbf{fgvc}\boldsymbol{=}\left( {\hat{\mathbf{w}}}^{s} \right)^{-1}{\hat{\mathbf{v}}}^{s}\mathbf{L}^{ss}\sum_{r\neq s}^{n} \mathbf{A}^{sr}\sum_{u}^{n} \mathbf{B}^{ru}\boldsymbol{(}\sum_{g}^{n} \mathbf{f}^{ug}\boldsymbol{)}$, (A5)

where the numerator is the third term in equation (A4).

The backward GVC participation indicator is constructed from the decomposition of final products. The value of a final product is equal to the summation of value-added contributions by all country-sectors. We decompose the final products of country $s$ as

${\boldsymbol{(}\mathbf{f}^{s}\boldsymbol{)}}^{\boldsymbol{'}}\boldsymbol{=}(\mathbf{v}^{s})'\mathbf{L}^{ss}{\hat{\mathbf{f}}}^{ss}\boldsymbol{+}(\mathbf{v}^{s})'\mathbf{L}^{ss}\sum_{r\neq s}^{n} {\hat{\mathbf{f}}}^{sr}\boldsymbol{+} \sum_{r}^{n} (\mathbf{v}^{r})'\mathbf{L}^{rr}\sum_{u\neq r}^{n} \mathbf{A}^{ur}\mathbf{B}^{rs}\sum_{g}^{n} {\hat{\mathbf{f}}}^{sg}$. (A6)

This decomposition gives the contribution of value-added from all source country-sectors to country $s$’s sectoral final products. The first term represents final products produced with only domestic value-added and consumed domestically. The second term refers to final products produced with only domestic value-added but consumed directly by the importing country $r$. This is traditional final products trade and it does not involve any foreign production activities either. The third term refers to all value-added that is embodied in country $s$’s final products and that includes border-crossing production activities. These are the foreign value-added in the imported inputs but also the domestic value-added that is in exported intermediate products which return in a next stage when they are embodied in imports.

The backward GVC participation index ($\mathbf{bgvc}$) can be calculated as the share of a country-sector’s final products produced with value-added involved in GVC activities, namely

$\mathbf{bgvc}\boldsymbol{=}\boldsymbol{\iota}^{'}\boldsymbol{-}\left( \mathbf{v}^{s} \right)^{'}\mathbf{L}^{ss}\left( {\hat{\mathbf{f}}}^{ss}\boldsymbol{+}\sum_{r\neq s}^{n} {\hat{\mathbf{f}}}^{sr} \right)\left( {\hat{\mathbf{f}}}^{s} \right)^{-1}=\boldsymbol{\iota}^{'}-\left( \mathbf{v}^{s} \right)^{'}\mathbf{L}^{ss}$. (A7)

A more traditional measure of GVC participation dates back to the vertical specialization (VS) and VS1 proposed by Hummels et al. (2001)^3^. VS refers to the imported foreign content in a country’s exports (backward participation), and VS1 calculates the value of a country’s intermediate exports that are used as inputs into another country’s production of export goods (forward participation). We can also use VS and VS1 to examine the robustness of the regression results.

Future research may also explore the relationship between GVC position and emission intensity. Some work has developed several measures of the positioning of countries and industries in GVCs. The often-adopted measures include the upstreamness and downstreamness^4^, and the average propagation length^5^. The “upstreamness” measures the distance of a country-industry from final use, and the “downstreamness” measures the distance from primary factors. Other things equal, it seems plausible that production processes that embody a larger number of intermediate inputs relative to their use of primary factors of production will be relatively downstream in value chains. APL is formulated as a weighted average of the number of production stages that an impact from industry A to industry E goes through, using the share of an impact at each stage as a weight. GVC position is associated with GVC participation, but they have different economic interpretations. Considering both the association and difference between GVC position and GVC participation, future research may investigate whether GVC position affect emission performance and what influence channels are.

**Measure the effects of GVC participation on emission intensity**

To examine the effects of GVC participation on emission intensity, we estimate a panel fixed effects model for industry $i$ of country $r$ at time $t$:

${emit}_{i,r,t}=\alpha+{\beta_{1}gvc}_{i,r,t}+\alpha_{i,r}\boldsymbol{+}\alpha_{i,t}\boldsymbol{+}\alpha_{r,t}\boldsymbol{+}\varepsilon_{i,r,t}$ . (A8)

The emission intensity (${emit}_{i,r,t}$) is the dependent variable. GVC participation (${gvc}_{i,r,t}$) is proxied by either forward participation (${fgvc}_{i,r,t}$) or backward participation (${bgvc}_{i,r,t}$). We include a set of industry-country fixed effects ($\alpha_{i,r}$), industry-year fixed effects ($\alpha_{i,t}$) and country-year fixed effects ($\alpha_{r,t}$). Note that we have accounted for possible country, industry and time unobserved heterogeneity by using such fixed effects, and thus, we argue that omitted variables bias is not a (big) concern.

To examine whether the effects of GVC participation on emission intensity are heterogeneous between developed countries and developing countries, we include in model (A8) an interaction between the stage of development ($d_{r}$) and the GVC participation indicators:

${emit}_{i,r,t}=\alpha+{\beta_{1}gvc}_{i,r,t}\boldsymbol{+}{\beta_{2}{gvc}_{i,r,t}*d}_{r}+\alpha_{i,r}\boldsymbol{+}\alpha_{i,t}\boldsymbol{+}\alpha_{r,t}\boldsymbol{+}\varepsilon_{i,r,t}$ . (A9)

The dummy variable $d_{r}$ denotes the stage of development. It is 1 for developed countries and 0 for developing countries. To classify the countries, we use the classification of the World Bank based on gross national income (GNI) per capita. Developed countries refer to as high-income economies, and developing countries refer to as low- and middle-income economies. In the empirical analysis, we also split the sample and run regressions on developed and developing countries separately. It turns out that the results based on the split samples tell the same story as our interaction model. We thereby present the results using the interaction model (A9).

We would like to stress that our main goal is to examine the association between GVC participation and emission intensity rather than causal effects. The regression model is potentially plagued by reverse causality. Lower level of emission intensity (i.e., cleaner production) may provide more opportunities for deeper integration into GVCs. For this reason, we refrain from stating any causal relationships.

We use the ICIO tables from the 2018 edition of the OECD Input-Output Database to calculate the forward and backward participation for 36 industries in 64 economies for the period 2005-2015 (11 years). As introduced above, the sectoral emission data is from the International Energy Agency. We can also use data from World Input-Output Database (WIOD)^6^ to check the robustness. WIOD (2013 edition) provides ICIO tables covering 35 sectors for 40 countries for the period 1995-2011, and sectoral emission data for the period 1995-2009.

**Supplementary References**

1. Tian, K.L., Dietzenbacher, E. & Jong-A-Pin, R. Global value chain participation and its impact on industrial upgrading. World Econ. 00, 1–24 (2021).
2. Wang, Z., Wei, S., Yu, X. & Zhu, K. Measures of participation in global value chains and global business cycles. NBER Working Paper No. 23222 (2017).
3. Hummels, D., Ishii, J. & Yi, K.M. The nature and growth of vertical specialization in world trade. J. Int. Econ. 54(1), 75–96 (2001).
4. Antràs, P., Chor, D., Fally, T. & Hillberry, R. Measuring the upstreamness of production and trade flows. Am. Econ. Rev. 102(3), 412–416 (2012).
5. Dietzenbacher, E. & Romero. I. Production chains in an interregional framework: identification by means of average propagation lengths. Int. Regional Sci. Rev. 30(4), 362–383 (2007).
6. Dietzenbacher, E., Los, B., Stehrer, R., Timmer, M. & de Vries, G. The construction of world input-output tables in the WIOD project. Econ. Syst. Res. 25, 71–98 (2013).


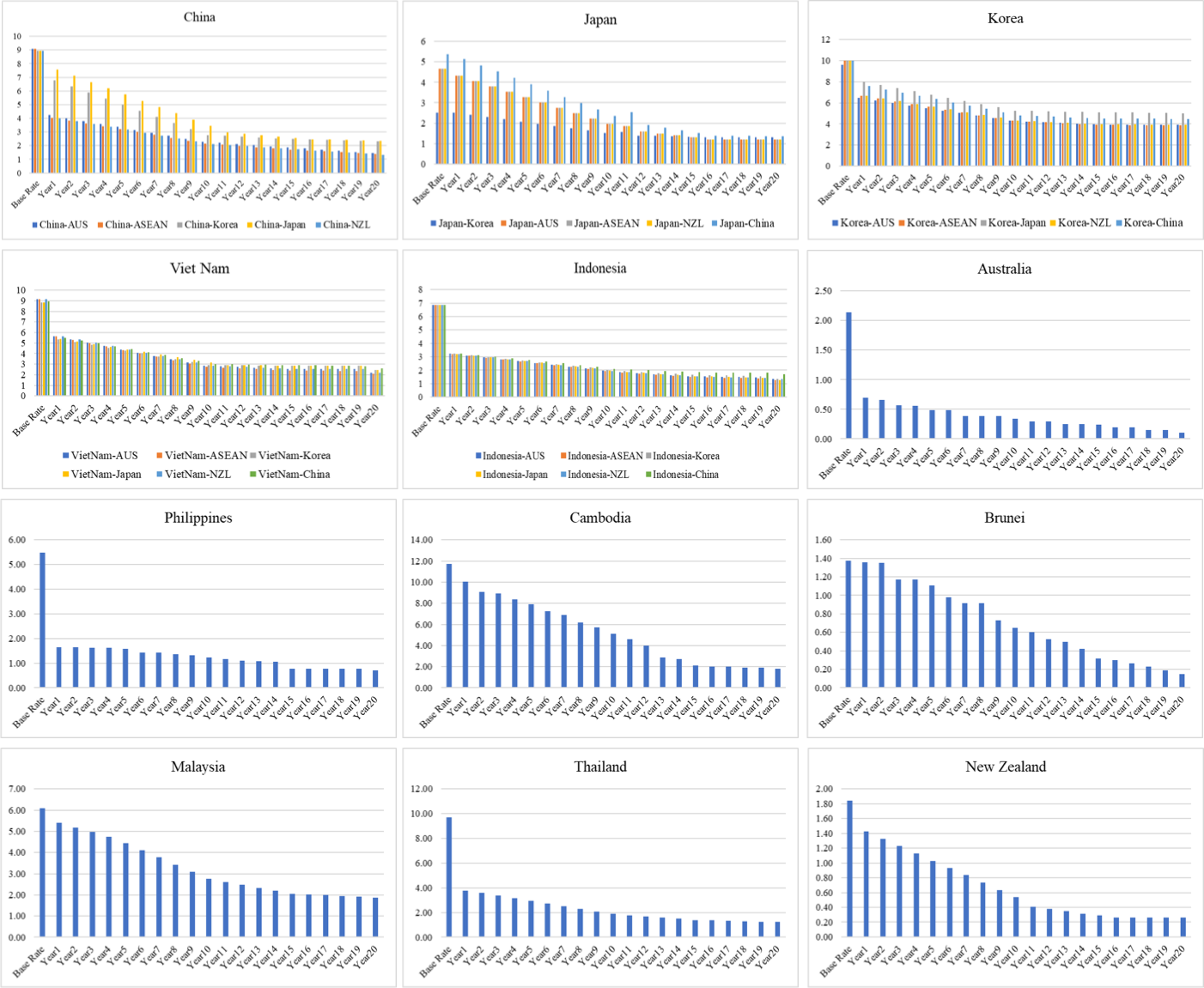


**Supplementary Figure 1.** The average import tariff imposed by RCEP members on other members after the RCEP agreement enters into force. The subfigures present the simple average import tariff imposed by RCEP members on other members after the RCEP agreement enters into force. We observe that the figures are different. For example, the figure for China and that for Australia appears to be very different. This means that China set different tariff schedules for different RCEP members (Japan, Korea, ASEAN, and others) in the RCEP agreement. Nevertheless, Australia set identical tariff schedules for all the RCEP members. As a result, we can observe several bars in the figure for some countries while one bar only for the rest of countries.

**Supplementary Table 1**

List of sectors in the 2018 edition of OECD Input-Output Database

| Number | Industry | Description |
| --- | --- | --- |
| 1 | Agriculture | Agriculture, forestry and fishing |
| 2 | Mining energy | Mining and extraction of energy producing products |
| 3 | Other mining | Mining and quarrying of non-energy producing products |
| 4 | Mining services | Mining support service activities |
| 5 | Food | Food products, beverages and tobacco |
| 6 | Textile | Textiles, wearing apparel, leather and related products |
| 7 | Wood | Wood and products of wood and cork |
| 8 | Paper | Paper products and printing |
| 9 | Petroleum | Coke and refined petroleum products |
| 10 | Chemicals | Chemicals and pharmaceutical products |
| 11 | Plastic | Rubber and plastic products |
| 12 | Minerals | Other non-metallic mineral products |
| 13 | Basic metals | Basic metals |
| 14 | Metal products | Fabricated metal products |
| 15 | Com | Computer, electronic and optical products |
| 16 | Electrical | Electrical equipment |
| 17 | Machinery nec | Machinery and equipment, nec |
| 18 | Motor | Motor vehicles, trailers and semi-trailers |
| 19 | Other Transport | Other transport equipment |
| 20 | Other | Other manufacturing; repair, installation of machinery and equipment |
| 21 | Energy supply | Electricity, gas, water supply, sewerage, and remediation services |
| 22 | Construction | Construction |
| 23 | Retail | Wholesale and retail trade; repair of motor vehicles |
| 24 | Transportation | Transportation and storage |
| 25 | Accommodation | Accommodation and food services |
| 26 | Publishing | Publishing, audiovisual and broadcasting activities |
| 27 | Telecommunications | Telecommunications |
| 28 | IT | IT and other information services |
| 29 | Finance | Financial and insurance activities |
| 30 | Real estate | Real estate activities |
| 31 | Other business | Other business sector services |
| 32 | Public | Public admin. and defence; compulsory social security |
| 33 | Education | Education |
| 34 | Health | Human health and social work |
| 35 | Entertainment | Arts, entertainment, recreation and other service activities |
| 36 | Private | Private households with employed persons |

**Supplementary Table 2**

The stylized environmentally-extended inter-country input-output table

|  | Intermediate use | | | | | Final use | | | | | Gross |
| --- | --- | --- | --- | --- | --- | --- | --- | --- | --- | --- | --- |
|  | in 1 | $\boldsymbol{\cdots}$ | in *r* | $\boldsymbol{\cdots}$ | in *n* | in 1 | $\boldsymbol{\cdots}$ | in *r* | $\boldsymbol{\cdots}$ | in *n* | outputs |
| Country 1 | $\mathbf{Z}^{11}$ | $\boldsymbol{\cdots}$ | $\mathbf{Z}^{1r}$ | $\boldsymbol{\cdots}$ | $\mathbf{Z}^{1n}$ | $\mathbf{f}^{11}$ | $\boldsymbol{\cdots}$ | $\mathbf{f}^{1r}$ | $\boldsymbol{\cdots}$ | $\mathbf{f}^{1n}$ | $\mathbf{y}^{1}$ |
| $\boldsymbol{\vdots}$ | $\boldsymbol{\vdots}$ | $\boldsymbol{\ddots}$ | $\boldsymbol{\vdots}$ | $\boldsymbol{⋰}$ | $\boldsymbol{\vdots}$ | $\boldsymbol{\vdots}$ | $\boldsymbol{\ddots}$ | $\boldsymbol{\vdots}$ | $\boldsymbol{⋰}$ | $\boldsymbol{\vdots}$ | $\boldsymbol{\vdots}$ |
| Country *r* | $\mathbf{Z}^{r1}$ | $\boldsymbol{\cdots}$ | $\mathbf{Z}^{rr}$ | $\boldsymbol{\cdots}$ | $\mathbf{Z}^{rn}$ | $\mathbf{f}^{r1}$ | $\boldsymbol{\cdots}$ | $\mathbf{f}^{rr}$ | $\boldsymbol{\cdots}$ | $\mathbf{f}^{rn}$ | $\mathbf{y}^{r}$ |
| $\boldsymbol{\vdots}$ | $\boldsymbol{\vdots}$ | $\boldsymbol{⋰}$ | $\boldsymbol{\vdots}$ | $\boldsymbol{\ddots}$ | $\boldsymbol{\vdots}$ | $\boldsymbol{\vdots}$ | $\boldsymbol{⋰}$ | $\boldsymbol{\vdots}$ | $\boldsymbol{\ddots}$ | $\boldsymbol{\vdots}$ | $\boldsymbol{\vdots}$ |
| Country *n* | $\mathbf{Z}^{n1}$ | $\boldsymbol{\cdots}$ | $\mathbf{Z}^{nr}$ | $\boldsymbol{\cdots}$ | $\mathbf{Z}^{nn}$ | $\mathbf{f}^{n1}$ | $\boldsymbol{\cdots}$ | $\mathbf{f}^{nr}$ | $\boldsymbol{\cdots}$ | $\mathbf{f}^{nn}$ | $\mathbf{y}^{n}$ |
| Value-added |  | $\boldsymbol{\cdots}$ |  | $\boldsymbol{\cdots}$ |  |  |  |  |  |  |  |
| Total inputs | $\boldsymbol{(}\mathbf{y}^{1}\boldsymbol{)'}$ | $\boldsymbol{\cdots}$ | $\boldsymbol{(}\mathbf{y}^{r}\boldsymbol{)'}$ | $\boldsymbol{\cdots}$ | $\boldsymbol{(}\mathbf{y}^{n}\boldsymbol{)'}$ |  |  |  |  |  |  |
| Emissions | $\boldsymbol{(}\mathbf{e}^{1}\boldsymbol{)'}$ |  | $\boldsymbol{(}\mathbf{e}^{r}\boldsymbol{)'}$ |  | $\boldsymbol{(}\mathbf{e}^{n}\boldsymbol{)'}$ |  |  |  |  |  |  |

**Supplementary Table 3**

Trade effects of RCEP’s tariff reductions in different situations: Zero tariff case (%)

|  | Australia | Brunei | China | Indonesia | Japan | Cambodia | Korea | Malaysia | New Zealand | Philippines | Singapore | Thailand | Vietnam |
| --- | --- | --- | --- | --- | --- | --- | --- | --- | --- | --- | --- | --- | --- |
| Australia |  | 10.08 | -9.63 | 148.90 | -2.54 | 192.87 | 18.25 | 31.09 | 2.79 | 77.31 | -4.65 | -7.18 | 112.06 |
| Brunei | -25.64 |  | 106.57 | 24.96 | -18.19 | 26.53 | 105.49 | 15.81 | 15.10 | 0.87 | -14.82 | 47.03 | 166.88 |
| China | 5.68 | 18.14 |  | 8.96 | 17.61 | 25.08 | 33.85 | 41.84 | 4.70 | 7.52 | 9.09 | 8.10 | 9.04 |
| Indonesia | -13.12 | -9.03 | 109.75 |  | -11.94 | 21.89 | 118.79 | 18.45 | 5.03 | 23.37 | -16.96 | 91.41 | 103.03 |
| Japan | -3.99 | 1.59 | 29.14 | -0.12 |  | 21.17 | 58.62 | 22.03 | 10.51 | -7.11 | -11.08 | -0.85 | -6.14 |
| Cambodia | -6.60 | -18.53 | 54.89 | 144.43 | 24.27 |  | 89.52 | 17.04 | 3.39 | 33.93 | -10.17 | 104.60 | 115.42 |
| Korea | 24.62 | 5.64 | 21.70 | 34.53 | 38.84 | 34.32 |  | 52.68 | 23.53 | 14.20 | 14.83 | 13.99 | 18.59 |
| Malaysia | -24.41 | 0.98 | 64.37 | 66.44 | 0.00 | 14.00 | 94.37 |  | -12.22 | 24.33 | -20.42 | 61.09 | 97.91 |
| New Zealand | -10.00 | 2.66 | -0.96 | 91.41 | 26.84 | 186.93 | 220.05 | 31.47 |  | 93.96 | -19.77 | -15.95 | 36.81 |
| Philippines | -1.26 | 9.84 | 46.06 | 49.68 | 4.42 | 28.01 | 175.91 | 24.64 | 17.08 |  | -10.01 | 77.76 | 82.07 |
| Singapore | -20.03 | -12.43 | 1.19 | 90.51 | -10.07 | 16.51 | -3.46 | 3.27 | -4.67 | -2.89 |  | 37.56 | 58.04 |
| Thailand | 4.44 | 7.62 | 56.58 | 65.57 | 9.40 | 36.38 | 122.99 | 29.62 | 4.80 | 37.99 | -9.41 |  | 99.25 |
| Vietnam | -4.57 | 1.94 | 50.74 | 111.46 | -1.12 | 28.51 | -15.10 | 27.97 | 14.69 | 52.54 | -9.77 | 166.13 |  |

Notes: The table presents the change rates in multi-lateral trade for the situation in which trade in goods among the RCEP members have zero tariffs. The results in this table are the same as that presented in Fig.1.

**Supplementary Table 4**

The change rates in multi-lateral trade: Year 1 (%)

|  | Australia | Brunei | China | Indonesia | Japan | Cambodia | Korea | Malaysia | New Zealand | Philippines | Singapore | Thailand | Vietnam |
| --- | --- | --- | --- | --- | --- | --- | --- | --- | --- | --- | --- | --- | --- |
| Australia |  | 7.19 | -5.16 | 104.61 | -0.95 | 60.71 | -7.49 | 16.48 | 0.14 | 46.02 | -2.55 | -7.58 | 80.17 |
| Brunei | -16.73 |  | 68.58 | 15.45 | -12.73 | 9.20 | 54.78 | 11.49 | -1.17 | 5.97 | -13.51 | 41.40 | 139.88 |
| China | 2.78 | 11.28 |  | 3.03 | 1.65 | 5.73 | -1.85 | 11.54 | 1.72 | 1.68 | 1.52 | -0.68 | 5.00 |
| Indonesia | -7.29 | -0.91 | 68.52 |  | -8.75 | 1.27 | 58.14 | 5.46 | -3.19 | 16.58 | -11.98 | 57.47 | 66.89 |
| Japan | 1.15 | 3.05 | -1.37 | 0.29 |  | 4.05 | 20.27 | 4.21 | 2.59 | -1.95 | -6.53 | -1.34 | -0.21 |
| Cambodia | -8.19 | -8.03 | 10.89 | 80.66 | 21.72 |  | 61.87 | 0.60 | -8.92 | 22.14 | -10.86 | 51.70 | 49.94 |
| Korea | 11.75 | 2.95 | -1.05 | 8.79 | 3.28 | 4.93 |  | 16.08 | 4.50 | 2.97 | 3.78 | 0.20 | 1.59 |
| Malaysia | -18.37 | -0.81 | 35.57 | 51.59 | 0.00 | -5.87 | 48.93 |  | -11.16 | 15.50 | -19.76 | 37.50 | 58.72 |
| New Zealand | -0.10 | 9.99 | -3.92 | 64.28 | -0.87 | 66.26 | -4.66 | 19.08 |  | 57.29 | -4.41 | -11.82 | 2.06 |
| Philippines | -0.08 | 7.44 | 29.59 | 23.49 | -0.59 | 6.38 | 54.68 | 5.80 | 1.23 |  | -5.47 | 45.26 | 32.12 |
| Singapore | -16.31 | -10.07 | -5.34 | 63.21 | -11.64 | -5.02 | -13.42 | -6.59 | -7.08 | -0.11 |  | 22.55 | 36.96 |
| Thailand | 0.95 | 10.35 | 27.82 | 29.07 | 0.03 | 12.00 | 39.05 | 7.98 | 0.73 | 20.15 | -3.47 |  | 57.67 |
| Vietnam | -2.46 | 5.08 | 27.60 | 73.83 | -3.53 | 9.94 | -9.43 | 8.03 | -2.26 | 37.99 | -3.38 | 94.61 |  |

Notes: The table presents the change rates in multi-lateral trade for the case in which the tariffs decline to the committed level in year 1 after the RCEP enters into force.

**Supplementary Table 5**

The change rates in multi-lateral trade: Year 5 (%)

|  | Australia | Brunei | China | Indonesia | Japan | Cambodia | Korea | Malaysia | New Zealand | Philippines | Singapore | Thailand | Vietnam |
| --- | --- | --- | --- | --- | --- | --- | --- | --- | --- | --- | --- | --- | --- |
| Australia |  | 8.59 | -5.92 | 117.12 | -0.46 | 86.93 | -9.04 | 21.07 | 0.05 | 47.88 | -1.79 | -7.25 | 85.29 |
| Brunei | -19.34 |  | 78.32 | 17.79 | -14.11 | 12.47 | 68.10 | 12.99 | 1.75 | 4.04 | -14.45 | 44.09 | 138.43 |
| China | 3.29 | 13.23 |  | 3.77 | 3.86 | 9.85 | -1.49 | 17.46 | 1.77 | 3.15 | 3.37 | -0.86 | 5.44 |
| Indonesia | -9.37 | -3.22 | 78.03 |  | -10.60 | 5.19 | 70.58 | 8.24 | -1.38 | 15.69 | -13.39 | 65.01 | 74.22 |
| Japan | 0.44 | 3.19 | 0.62 | -0.02 |  | 8.09 | 30.31 | 7.70 | 4.09 | -2.44 | -6.89 | -1.76 | -1.05 |
| Cambodia | -8.93 | -10.62 | 20.73 | 90.01 | 18.60 |  | 69.83 | 2.88 | -8.07 | 23.54 | -10.82 | 64.16 | 66.75 |
| Korea | 14.82 | 3.62 | -1.68 | 13.00 | 5.34 | 9.29 |  | 23.79 | 5.85 | 5.68 | 7.06 | 0.98 | 2.16 |
| Malaysia | -20.68 | -0.79 | 44.60 | 52.90 | 0.00 | -2.51 | 58.67 |  | -12.94 | 15.38 | -20.76 | 43.69 | 67.32 |
| New Zealand | -0.27 | 11.65 | -4.57 | 74.30 | -0.33 | 94.32 | -1.42 | 24.36 |  | 59.37 | -3.84 | -12.91 | 3.22 |
| Philippines | -1.18 | 7.08 | 35.53 | 27.79 | -1.96 | 9.49 | 60.19 | 8.42 | 1.75 |  | -6.97 | 52.91 | 48.94 |
| Singapore | -17.29 | -10.52 | -3.92 | 68.78 | -12.08 | -0.66 | -12.65 | -4.44 | -7.48 | -0.82 |  | 27.97 | 38.63 |
| Thailand | 0.96 | 10.02 | 35.37 | 33.61 | -0.29 | 17.67 | 46.59 | 11.29 | 0.43 | 21.28 | -4.42 |  | 69.47 |
| Vietnam | -4.21 | 3.85 | 34.97 | 78.55 | -5.30 | 13.30 | -12.95 | 10.77 | 0.13 | 37.26 | -6.19 | 111.60 |  |

Notes: The table presents the change rates in multi-lateral trade for the case in which the tariffs decline to the committed level in year 5 after the RCEP enters into force.

**Supplementary Table 6**

The change rates in multi-lateral trade: Year 10 (%)

|  | Australia | Brunei | China | Indonesia | Japan | Cambodia | Korea | Malaysia | New Zealand | Philippines | Singapore | Thailand | Vietnam |
| --- | --- | --- | --- | --- | --- | --- | --- | --- | --- | --- | --- | --- | --- |
| Australia |  | 10.16 | -7.30 | 132.48 | 0.48 | 128.32 | -10.14 | 27.06 | -0.07 | 52.68 | -1.42 | -7.41 | 95.78 |
| Brunei | -22.38 |  | 88.62 | 20.73 | -15.87 | 16.84 | 88.20 | 14.80 | 5.47 | 1.77 | -16.04 | 46.44 | 134.14 |
| China | 4.19 | 16.01 |  | 4.92 | 8.33 | 15.57 | 4.88 | 26.91 | 2.03 | 5.10 | 6.07 | 1.71 | 4.81 |
| Indonesia | -11.74 | -6.00 | 88.79 |  | -12.49 | 10.58 | 88.78 | 12.58 | 1.17 | 15.51 | -15.17 | 74.26 | 84.19 |
| Japan | -1.66 | 2.75 | 10.36 | -0.71 |  | 13.09 | 42.51 | 12.00 | 5.35 | -4.69 | -8.61 | -2.80 | -4.13 |
| Cambodia | -9.85 | -13.92 | 34.50 | 103.15 | 15.22 |  | 76.56 | 11.41 | -6.10 | 27.71 | -10.75 | 80.15 | 91.49 |
| Korea | 17.68 | 4.24 | 1.23 | 18.02 | 8.87 | 15.12 |  | 33.67 | 6.66 | 8.94 | 9.82 | 4.19 | 7.78 |
| Malaysia | -22.74 | -0.38 | 55.34 | 55.76 | 0.00 | 4.28 | 72.62 |  | -14.65 | 17.00 | -21.44 | 51.48 | 80.17 |
| New Zealand | -0.76 | 13.06 | -5.74 | 87.09 | 1.28 | 138.34 | 7.87 | 31.31 |  | 65.47 | -4.03 | -15.83 | 20.84 |
| Philippines | -2.16 | 7.10 | 42.36 | 34.33 | -2.61 | 14.66 | 66.90 | 13.16 | 4.33 |  | -8.34 | 62.18 | 70.13 |
| Singapore | -18.30 | -11.27 | -2.18 | 75.46 | -12.26 | 5.20 | -10.68 | -1.49 | -7.88 | -1.36 |  | 33.99 | 42.97 |
| Thailand | 1.20 | 9.87 | 45.21 | 40.34 | 0.21 | 25.89 | 56.68 | 17.40 | 0.41 | 24.23 | -5.34 |  | 85.84 |
| Vietnam | -5.82 | 2.38 | 43.80 | 85.38 | -6.69 | 17.29 | -18.97 | 16.25 | 4.47 | 38.79 | -9.28 | 133.17 |  |

Notes: The table presents the change rates in multi-lateral trade for the case in which the tariffs decline to the committed level in year 10 after the RCEP enters into force.

**Supplementary Table 7**

The change rates in multi-lateral trade: Year 20 (%)

|  | Australia | Brunei | China | Indonesia | Japan | Cambodia | Korea | Malaysia | New Zealand | Philippines | Singapore | Thailand | Vietnam |
| --- | --- | --- | --- | --- | --- | --- | --- | --- | --- | --- | --- | --- | --- |
| Australia |  | 11.14 | -8.03 | 141.51 | 0.15 | 178.27 | -9.79 | 30.43 | -0.04 | 65.61 | -1.31 | -6.16 | 101.37 |
| Brunei | -23.16 |  | 98.71 | 22.00 | -16.87 | 21.46 | 92.31 | 17.72 | 9.55 | 2.43 | -16.53 | 47.53 | 134.32 |
| China | 4.66 | 17.38 |  | 4.98 | 11.54 | 23.47 | 7.11 | 31.93 | 2.17 | 6.43 | 7.58 | 4.66 | 5.28 |
| Indonesia | -12.46 | -7.15 | 97.18 |  | -13.20 | 17.21 | 90.81 | 14.28 | 2.88 | 17.99 | -15.98 | 78.54 | 89.65 |
| Japan | -2.22 | 2.47 | 16.63 | -1.39 |  | 18.90 | 44.49 | 14.97 | 6.81 | -5.83 | -9.20 | -2.79 | -5.13 |
| Cambodia | -6.19 | -17.10 | 47.94 | 131.39 | 17.70 |  | 85.39 | 14.45 | -2.66 | 33.85 | -10.17 | 89.34 | 103.00 |
| Korea | 17.57 | 4.24 | 2.17 | 19.20 | 15.73 | 21.54 |  | 38.01 | 6.48 | 10.69 | 9.67 | 6.61 | 10.32 |
| Malaysia | -23.76 | -0.02 | 62.43 | 58.93 | 0.00 | 11.48 | 75.23 |  | -15.57 | 20.40 | -21.83 | 54.71 | 84.51 |
| New Zealand | -1.28 | 13.55 | -6.51 | 94.30 | 2.26 | 192.30 | 11.64 | 33.08 |  | 83.12 | -4.60 | -17.47 | 30.84 |
| Philippines | -1.65 | 8.89 | 48.81 | 42.39 | -2.19 | 23.66 | 74.21 | 17.23 | 8.23 |  | -7.67 | 69.07 | 77.91 |
| Singapore | -19.03 | -12.00 | 0.10 | 78.48 | -12.64 | 10.93 | -9.95 | 0.62 | -8.05 | -1.14 |  | 36.31 | 43.23 |
| Thailand | 1.53 | 9.59 | 51.12 | 47.70 | 0.44 | 34.30 | 60.69 | 21.85 | 0.47 | 28.84 | -5.98 |  | 92.18 |
| Vietnam | -6.31 | 1.60 | 48.64 | 95.08 | -7.42 | 23.37 | -20.39 | 18.13 | 7.35 | 44.82 | -11.16 | 140.31 |  |

Notes: The table presents the change rates in multi-lateral trade for the case in which the tariffs decline to the committed level in year 20 after the RCEP enters into force.

**Supplementary Table 8**

Welfare effects of RCEP’s tariff reductions in different situations (%)

| Economy | Year 1 | | | | Year 5 | | | | Year 10 | | | |
| --- | --- | --- | --- | --- | --- | --- | --- | --- | --- | --- | --- | --- |
|  | Welfare | Term of trade | Volume of trade | Real wage | Welfare | Term of trade | Volume of trade | Real wage | Welfare | Term of trade | Volume of trade | Real wage |
| Argentina | 0.00 | 0.00 | 0.00 | 0.00 | 0.00 | 0.00 | 0.00 | 0.00 | 0.00 | 0.00 | 0.00 | 0.00 |
| Brazil | -0.03 | -0.03 | 0.00 | -0.03 | -0.03 | -0.03 | 0.00 | -0.03 | -0.03 | -0.03 | 0.00 | -0.03 |
| Canada | 0.02 | 0.02 | 0.00 | -0.02 | 0.03 | 0.03 | 0.00 | -0.02 | 0.06 | 0.06 | 0.00 | -0.02 |
| Switzerland | -0.02 | -0.02 | 0.00 | -0.03 | -0.02 | -0.02 | 0.00 | -0.03 | 0.00 | 0.00 | 0.00 | -0.03 |
| Chile | -0.03 | -0.03 | 0.00 | -0.01 | -0.03 | -0.03 | 0.00 | -0.01 | -0.03 | -0.02 | 0.00 | -0.01 |
| Colombia | 0.04 | 0.04 | 0.00 | 0.00 | 0.06 | 0.06 | 0.00 | 0.01 | 0.09 | 0.09 | 0.00 | 0.00 |
| Costa Rica | -0.05 | -0.04 | 0.00 | -0.03 | -0.05 | -0.04 | 0.00 | -0.03 | -0.06 | -0.05 | 0.00 | -0.03 |
| EU | 0.02 | 0.02 | 0.00 | 0.02 | 0.02 | 0.02 | 0.00 | 0.02 | 0.02 | 0.02 | 0.00 | 0.02 |
| India | -0.03 | -0.02 | 0.00 | -0.02 | -0.03 | -0.02 | -0.01 | -0.02 | -0.04 | -0.03 | -0.01 | -0.02 |
| Iceland | -0.08 | -0.23 | 0.14 | -0.16 | -0.08 | -0.23 | 0.14 | -0.16 | -0.09 | -0.24 | 0.15 | -0.16 |
| Israel | 0.01 | 0.01 | 0.00 | 0.02 | 0.00 | 0.00 | 0.00 | 0.01 | -0.02 | -0.01 | 0.00 | 0.02 |
| Morocco | 0.00 | 0.00 | 0.00 | 0.00 | -0.01 | 0.00 | 0.00 | 0.00 | -0.03 | -0.01 | -0.01 | 0.00 |
| Mexico | -0.01 | -0.01 | 0.00 | -0.02 | 0.00 | 0.00 | 0.00 | -0.02 | 0.00 | 0.00 | 0.00 | -0.02 |
| Norway | -0.03 | -0.03 | 0.00 | -0.03 | -0.03 | -0.03 | 0.00 | -0.03 | -0.03 | -0.03 | 0.00 | -0.03 |
| Russia | -0.12 | -0.12 | 0.00 | -0.03 | -0.12 | -0.13 | 0.00 | -0.03 | -0.14 | -0.14 | 0.00 | -0.03 |
| Saudi Arabia | -0.02 | -0.03 | 0.00 | 0.00 | -0.02 | -0.02 | 0.00 | 0.00 | -0.01 | -0.02 | 0.00 | 0.00 |
| Turkey | -1.09 | -0.08 | -1.01 | -0.91 | -1.08 | -0.07 | -1.01 | -0.90 | -1.04 | -0.03 | -1.01 | -0.91 |
| United States | -0.01 | -0.01 | 0.00 | -0.01 | -0.01 | -0.01 | 0.00 | -0.01 | -0.02 | -0.02 | 0.00 | -0.01 |
| South Africa | 0.02 | 0.02 | 0.00 | 0.02 | 0.03 | 0.03 | 0.00 | 0.03 | 0.05 | 0.05 | 0.00 | 0.02 |
| Australia | 0.03 | 0.03 | 0.00 | 0.04 | 0.04 | 0.04 | 0.00 | 0.05 | 0.06 | 0.06 | 0.00 | 0.04 |
| Brunei | 0.30 | 0.30 | 0.00 | 0.20 | 0.37 | 0.37 | 0.00 | 0.26 | 0.47 | 0.47 | 0.00 | 0.20 |
| China | 0.08 | -0.07 | 0.15 | 0.03 | 0.12 | -0.09 | 0.21 | 0.07 | 0.15 | -0.13 | 0.28 | 0.13 |
| Hong Kong | -0.08 | -0.08 | 0.00 | -0.09 | -0.06 | -0.06 | 0.00 | -0.10 | -0.02 | -0.02 | 0.00 | -0.09 |
| Indonesia | 0.37 | 0.19 | 0.18 | 0.37 | 0.46 | 0.25 | 0.21 | 0.44 | 0.57 | 0.32 | 0.24 | 0.37 |
| Japan | 0.03 | 0.03 | 0.00 | 0.02 | 0.06 | 0.06 | 0.00 | 0.05 | 0.15 | 0.13 | 0.02 | 0.02 |
| Cambodia | 4.79 | 3.63 | 1.16 | 2.25 | 5.65 | 3.80 | 1.86 | 3.96 | 6.78 | 3.92 | 2.86 | 2.25 |
| Korea | 0.08 | -0.16 | 0.24 | 0.19 | 0.06 | -0.27 | 0.33 | 0.26 | 0.13 | -0.36 | 0.49 | 0.19 |
| Malaysia | 1.13 | 0.99 | 0.14 | 1.13 | 1.28 | 0.99 | 0.29 | 1.68 | 1.40 | 0.87 | 0.54 | 1.13 |
| New Zealand | 0.06 | 0.06 | 0.00 | 0.05 | 0.07 | 0.07 | 0.00 | 0.07 | 0.09 | 0.09 | 0.00 | 0.05 |
| Philippines | 0.13 | -0.09 | 0.22 | 0.72 | 0.18 | -0.05 | 0.23 | 0.84 | 0.24 | -0.02 | 0.27 | 0.72 |
| Singapore | 2.76 | 2.76 | 0.00 | 2.54 | 2.97 | 2.97 | 0.00 | 2.74 | 3.27 | 3.27 | 0.00 | 2.54 |
| Thailand | 0.74 | -0.21 | 0.96 | 1.04 | 0.97 | -0.21 | 1.18 | 1.33 | 1.22 | -0.28 | 1.50 | 1.04 |
| Vietnam | 9.55 | 4.57 | 4.98 | 1.78 | 11.69 | 5.82 | 5.87 | 2.24 | 13.79 | 6.49 | 7.30 | 1.78 |

**Supplementary Table 9**

Welfare effects of RCEP’s tariff reductions in different situations (continued, %)

| Economy | Year 20 | | | | Zero tariff | | | |
| --- | --- | --- | --- | --- | --- | --- | --- | --- |
|  | Welfare | Term of trade | Volume of trade | Real wage | Welfare | Term of trade | Volume of trade | Real wage |
| Argentina | 0.01 | 0.01 | 0.00 | 0.01 | 0.01 | 0.00 | 0.00 | 0.02 |
| Brazil | -0.03 | -0.02 | -0.01 | -0.02 | -0.02 | -0.02 | 0.00 | -0.02 |
| Canada | 0.07 | 0.07 | 0.00 | -0.01 | 0.12 | 0.12 | 0.00 | 0.01 |
| Switzerland | 0.01 | 0.01 | 0.00 | -0.04 | 0.04 | 0.04 | 0.00 | -0.04 |
| Chile | -0.03 | -0.02 | 0.00 | 0.01 | -0.04 | -0.03 | 0.00 | 0.02 |
| Colombia | 0.11 | 0.11 | 0.00 | 0.03 | 0.18 | 0.17 | 0.00 | 0.07 |
| Costa Rica | -0.07 | -0.06 | -0.01 | -0.03 | -0.09 | -0.08 | 0.00 | -0.04 |
| EU | 0.01 | 0.02 | 0.00 | 0.01 | 0.01 | 0.01 | 0.00 | 0.01 |
| India | -0.05 | -0.03 | -0.02 | -0.02 | -0.06 | -0.04 | -0.02 | -0.02 |
| Iceland | -0.10 | -0.24 | 0.15 | -0.16 | -0.14 | -0.28 | 0.14 | -0.17 |
| Israel | -0.03 | -0.02 | 0.00 | 0.00 | -0.07 | -0.06 | 0.00 | -0.02 |
| Morocco | -0.03 | -0.02 | -0.02 | 0.00 | -0.06 | -0.04 | -0.02 | -0.01 |
| Mexico | 0.01 | 0.01 | 0.00 | -0.02 | 0.04 | 0.04 | 0.00 | -0.01 |
| Norway | -0.02 | -0.02 | 0.00 | -0.02 | -0.01 | 0.00 | 0.00 | 0.00 |
| Russia | -0.15 | -0.15 | 0.00 | -0.03 | -0.15 | -0.15 | 0.00 | -0.01 |
| Saudi Arabia | -0.01 | -0.01 | 0.00 | 0.02 | 0.04 | 0.03 | 0.00 | 0.05 |
| Turkey | -1.02 | -0.02 | -1.01 | -0.90 | -0.96 | 0.05 | -1.01 | -0.87 |
| United States | -0.02 | -0.02 | 0.00 | -0.01 | -0.02 | -0.02 | 0.00 | -0.01 |
| South Africa | 0.07 | 0.06 | 0.00 | 0.06 | 0.11 | 0.11 | 0.00 | 0.10 |
| Australia | 0.07 | 0.07 | 0.00 | 0.10 | 0.20 | 0.20 | 0.00 | 0.25 |
| Brunei | 0.50 | 0.50 | 0.00 | 0.37 | 0.59 | 0.59 | 0.00 | 0.47 |
| China | 0.19 | -0.18 | 0.37 | 0.27 | 0.34 | 0.17 | 0.17 | 0.36 |
| Hong Kong | 0.00 | 0.00 | 0.00 | -0.14 | 0.11 | 0.11 | 0.00 | -0.12 |
| Indonesia | 0.63 | 0.36 | 0.28 | 0.62 | 0.81 | 0.42 | 0.39 | 0.88 |
| Japan | 0.19 | 0.16 | 0.03 | 0.17 | 0.28 | 0.23 | 0.05 | 0.28 |
| Cambodia | 7.24 | 3.15 | 4.09 | 9.55 | 8.54 | 3.88 | 4.66 | 11.86 |
| Korea | 0.23 | -0.34 | 0.57 | 0.55 | 3.28 | -0.70 | 3.98 | 2.38 |
| Malaysia | 1.47 | 0.78 | 0.69 | 3.27 | 1.31 | 0.26 | 1.05 | 4.68 |
| New Zealand | 0.11 | 0.11 | 0.01 | 0.13 | 0.47 | 0.46 | 0.02 | 0.38 |
| Philippines | 0.29 | -0.05 | 0.34 | 1.27 | 0.52 | 0.02 | 0.50 | 2.01 |
| Singapore | 3.49 | 3.49 | 0.00 | 3.23 | 3.79 | 3.79 | 0.00 | 3.52 |
| Thailand | 1.35 | -0.35 | 1.70 | 2.19 | 1.82 | -0.49 | 2.31 | 3.15 |
| Vietnam | 14.97 | 7.01 | 7.95 | 3.44 | 15.63 | 5.84 | 9.78 | 5.60 |

Notes: The table presents the change in welfare for the cases in which the tariffs decline to the committed level in year 1, 5, 10, 20, and ultimately zero after the RCEP enters into force.

**Supplementary Table 10**

Underlying data for Fig.3

|  | Year 1 | Year 5 | Year 10 | No tariff | Relative welfare/emission | Welfare change/emission change |
| --- | --- | --- | --- | --- | --- | --- |
| Australia | 3.71 | 4.96 | 12.29 | 24.04 | 0.032 | 112.88 |
| China | 117.27 | 253.69 | 382.71 | 495.73 | 0.065 | 61.36 |
| Japan | 13.22 | 32.07 | 42 | 52.66 | 0.057 | 255.52 |
| Korea | 13.92 | 20.85 | 30.99 | 50.34 | 0.395 | 838.38 |
| New Zealand | 0.49 | 0.72 | 1.09 | 1.56 | 0.095 | 554.78 |
| ASEAN | 59.65 | 83.73 | 136.93 | 164.72 |  |  |
| Brunei | 0.12 | 0.27 | 0.33 | 0.52 | 0.081 | 197.28 |
| Cambodia | 0.46 | 0.63 | 0.78 | 1.15 | 0.783 | 1168.19 |
| Indonesia | 14.27 | 23.51 | 40.71 | 48.53 | 0.091 | 144.98 |
| Malaysia | 12.8 | 18.26 | 28.23 | 36.7 | 0.081 | 119.37 |
| Philippines | 2.24 | 3.37 | 4.46 | 5.42 | 0.127 | 266.82 |
| Singapore | 0.23 | 0.56 | 1.55 | 2.26 | 0.794 | 4852.11 |
| Thailand | 11.1 | 13.74 | 25.76 | 32.74 | 0.134 | 213.33 |
| Vietnam | 18.43 | 23.39 | 35.11 | 37.4 | 0.947 | 707.51 |
| US | 2.17 | 3.33 | 6.81 | 15.59 |  |  |
| EU | 5.51 | 6.67 | 10.08 | 14.01 |  |  |
| RoW | 35.41 | 57.7 | 133.48 | 227.8 |  |  |
| Total | 251.35 | 463.72 | 756.38 | 1046.45 |  |  |

Notes: The first four columns present the emission changes (Mt CO_2_) in different situation. The fifth column presents the ratio which gives the welfare gains (%) at the cost of a 1% increase in carbon emissions. The last column presents the amount of welfare changes (million dollars) relative to the changes of carbon emissions (Mt).

**Supplementary Table 11**

Underlying data for Fig.4 (Mt CO_2_)

|  | China | ASEAN | Japan | South Korea | Australia | New Zealand | RoW |
| --- | --- | --- | --- | --- | --- | --- | --- |
| China |  | 44.28 | 36.07 | 27.83 | 9.72 | 8.03 | 10.12 |
| ASEAN | 40.24 |  | 5.65 | 16.26 | 5.30 | 4.60 | 2.05 |
| Japan | 9.59 | 4.96 |  | 3.40 | 0.99 | 0.66 | 0.80 |
| South Korea | 9.48 | 7.86 | 6.46 |  | 1.87 | 1.38 | 2.27 |
| Australia | 1.94 | 1.17 | 1.14 | 0.16 |  | 0.33 | 0.30 |
| New Zealand | 0.32 | 0.08 | 0.03 | 0.07 | 0.03 |  | 0.01 |
| RoW | 18.48 | 11.08 | 8.48 | 12.56 | 7.66 | 2.93 | 0 |

**Supplementary Table 12**

Underlying data for Fig.5 (%)

|  | China | ASEAN | Japan | South Korea | Australia | New Zealand |
| --- | --- | --- | --- | --- | --- | --- |
| Agriculture | 3.72 | 10.44 | 0.73 | 2.67 | 10.69 | 45.77 |
| Mining | 3.75 | 3.94 | 2.88 | 3.06 | 43.43 | 2.81 |
| Food | 3.65 | 4.17 | 0.41 | 0.99 | 2.25 | 13.44 |
| Textile | 9.37 | 3.99 | 1.39 | 2.67 | 1.14 | 1.83 |
| Wood | 3.02 | 3.00 | 0.05 | 1.02 | 2.56 | 6.87 |
| Paper | 4.00 | 4.61 | 0.57 | 1.76 | 2.29 | 2.92 |
| Petroleum | 2.69 | 4.77 | 4.34 | 8.38 | 8.07 | 3.04 |
| Chemicals | 5.80 | 4.05 | 8.72 | 7.52 | 4.46 | 4.97 |
| Plastic | 3.97 | 10.66 | 5.98 | 7.02 | 0.40 | 2.99 |
| Minerals | 4.84 | 8.07 | 2.95 | 1.04 | 9.71 | 2.40 |
| Basic metals | 4.49 | 3.43 | 8.80 | 8.42 | 5.40 | 2.72 |
| Metal products | 6.10 | 3.47 | 1.11 | 0.82 | 2.10 | 2.18 |
| Computer | 8.25 | 4.87 | 6.13 | 10.90 | 1.05 | 1.59 |
| Electrical | 14.53 | 12.73 | 18.72 | 23.65 | 1.60 | 1.37 |
| Machinery nec | 12.22 | 10.89 | 17.62 | 9.87 | 2.98 | 3.23 |
| Motor vehicles | 2.55 | 5.61 | 17.75 | 8.61 | 1.24 | 1.27 |
| Other transport | 2.43 | 0.10 | 0.75 | 1.01 | 0.42 | 0.23 |
| Other | 4.62 | 1.19 | 1.10 | 0.58 | 0.23 | 0.37 |
